# Proximity Induced Magnetic Anisotropy and Trefoil Fermiology in Monolayer FeCl_2_/Bi(111)

**DOI:** 10.1002/adma.202521534

**Published:** 2026-02-19

**Authors:** Shigemi Terakawa, Binbin Liu, Frank Schindler, Titus Neupert, Jing‐Rong Ji, Gabriele Domaine, Emily C. McFarlane, Daiyu Geng, Jiabao Yang, Fadi Choueikani, Philippe Ohresser, Manuel Valvidares, Pierluigi Gargiani, Craig Polley, Gerardina Carbone, Mats Leandersson, Stuart S. P. Parkin, Amilcar Bedoya‐Pinto, Niels B. M. Schröter

**Affiliations:** ^1^ Department of Applied Physics Graduate School of Engineering The University of Osaka Osaka Japan; ^2^ Center for Future Innovation Graduate School of Engineering The University of Osaka Osaka Japan; ^3^ Max Planck Institute of Microstructure Physics Halle Germany; ^4^ Theory and Simulations of Materials (THEOS), and National Centre for Computational Design and Discovery of Novel Materials (MARVEL) École Polytechnique Fédérale de Lausanne Lausanne Switzerland; ^5^ Blackett Laboratory Imperial College London London United Kingdom; ^6^ Department of Physics University of Zurich Zurich Switzerland; ^7^ Synchrotron SOLEIL, L'Orme des Merisiers Saint‐Aubin France; ^8^ ALBA Synchrotron Light Source Barcelona Spain; ^9^ MAX IV Laboratory Lund University Lund Sweden; ^10^ Institute of Molecular Science (ICMoL) University of Valencia Paterna Spain; ^11^ Institute of Physics Martin Luther University Halle‐Wittenberg and Halle‐Berlin‐Regensburg Cluster of Excellence CCE Halle Germany

**Keywords:** interface states, magnetic anisotropy, trefoil Fermi surface, vdW heterostructure, 2D magnet

## Abstract

Interfaces between magnetic and non‐magnetic materials play a crucial role in various magnetic heterostructures. The emergence of 2D van der Waals (vdW) magnets has introduced new opportunities for exploring proximity effects in vdW heterostructures. While the influence of magnetic layers on nearby non‐magnetic materials has been widely studied, it remains unclear whether non‐magnetic substrates can similarly modulate the intrinsic magnetic properties of 2D magnets, particularly their magnetic anisotropy. In this work, by analyzing X‐ray magnetic circular dichroism spectra of an epitaxially grown FeCl_2_ monolayer on a Bi(111) surface, a reorientation of magnetic anisotropy is observed – from its natural out‐of‐plane to a predominantly in‐plane alignment. This effect vanishes in bilayer FeCl_2_/Bi(111), where the magnetic anisotropy reverts to its intrinsic out‐of‐plane orientation, consistent with the layered antiferromagnetic order of bulk FeCl_2_. Angle‐resolved photoelectron spectroscopy reveals the presence of metallic interface states derived from the Bi surface states, accompanied by charge transfer and emergence of a moiré potential that gives rise to a distinctive trefoil‐shaped Fermi surface. These results demonstrate that non‐magnetic substrates can exert strong proximity influence on the magnetic and electronic behavior of 2D vdW magnets, offering new strategies for engineering magnetic anisotropy and electronic structure in spintronic heterostructures.

## Graphical abstract

Reorientation of magnetic anisotropy in a 2D van der Waals magnet is demonstrated in monolayer FeCl_2_ epitaxially grown on a bismuth substrate, from its natural out‐of‐plane direction to a predominantly in‐plane orientation. The FeCl_2_/bismuth heterostructure exhibits moiré‐induced interface states, suggesting strong coupling at the interface. These findings highlight new strategies for engineering magnetic anisotropy in spintronic heterostructures.

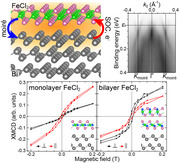

## 1 Introduction

2D van der Waals (vdW) magnetic materials serve as an ideal platform to investigate magnetism in the 2D limit and hold promise for next‐generation spintronic applications [1, 2, 3, 4, 5]. Heterostructures involving 2D vdW magnets can lead to topological superconductivity and the quantum anomalous Hall effect, in which the magnetic proximity effect from 2D magnets toward adjacent non‐magnetic materials plays a key role and has been extensively studied [6, 7]. In contrast, interfacial proximity effects from non‐magnetic materials on the magnetic properties of 2D vdW magnets are less investigated. Whilst there have been some studies on the change of magnetic transition temperature and the enhancement of magnetic anisotropy by proximity effects [8, 9, 10], to the best of our knowledge, no proximity‐induced reorientation of the magnetic anisotropy has been reported so far.

**FIGURE 1:**
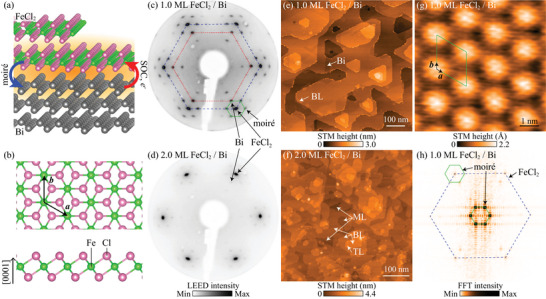
(a) Schematic illustration of the FeCl_2_/Bi heterostructure. (b) Top and side views of the atomic structure of the monolayer FeCl_2_. The atomic structures were visualized using VESTA [29]. LEED patterns of the (c) 1.0‐ML and (d) 2.0‐ML FeCl_2_ films on Bi(111) ($E_{p}$ = 46 eV). Large‐scale STM images of the (e) 1.0‐ML and (f) 2.0‐ML FeCl_2_ films on Bi(111). ML, BL and TL denote monolayer, bilayer and trilayer FeCl_2_, respectively. (g) High‐resolution STM image and (h) FFT pattern of the 1.0‐ML FeCl_2_ film on Bi(111). The images were obtained at (e) sample bias $V_{S}$ = +0.5 V and tunneling current I = 2 pA, (f) $V_{S}$ = +3.0 V and I = 2 pA, and (g) $V_{S}$ = +3.0 V and I = 2 nA. The LEED patterns and STM images were obtained at room temperature. The blue dashed, red dotted, and green solid hexagons in (c) and (h) indicate the spots corresponding to the lattices of FeCl_2_(0001), Bi(111), and moiré superstructure, respectively.

Recently, transition metal dihalides have received increasing attention as a new class of 2D magnets [11, 12, 13, 14, 15, 16, 17, 18, 19, 20, 21, 22]. Notably, monolayer NiI_2_ was found to be the first 2D material exhibiting multiferroic order [23, 24]. FeCl_2_ is an isomorphic cousin of NiI_2_ with a CdCl_2_‐type structure composed of Cl‐Fe‐Cl sandwich layers (Figure 1b) stacked along the [0001] direction by weak vdW interaction [25, 26]. Bulk FeCl_2_ is an Ising‐type antiferromagnetic (AFM) insulator with a Néel temperature of 24 K [25]. Its magnetic moments are ferromagnetically aligned along the [0001] out‐of‐plane direction and antiferromagnetically coupled between the adjacent layers. Bulk FeCl_2_ exhibits a magnetic‐field‐induced metamagnetic transition from AFM to a saturated paramagnetic state at around 1.5 T [27, 28].

Monolayer FeCl_2_ has been studied theoretically using density functional theory (DFT) calculations, but there is no consensus regarding its electronic properties. Although a half‐metallic electronic structure was initially predicted [30, 31, 32], recent theoretical studies predicted that the ground state becomes insulating due to electronic correlations, spin‐orbit coupling (SOC), and a trigonal distortion along the [0001] direction [33, 34, 35, 36, 37, 38]. The calculated magnetic ground state is also disputed. Some studies report a ferromagnetic (FM) state with an out‐of‐plane easy magnetization axis [33, 36], others a FM state with an easy plane [32, 34, 38], and a recent work predicts a helimagnetic ground state [37].

Very recently, the magnetic ground state of the monolayer FeCl_2_ grown on a Au(111) substrate has been reported to be FM with the out‐of‐plane easy axis [20, 21], which is consistent with the out‐of‐plane magnetic anisotropy of bulk FeCl_2_. However, the magnetic transition temperatures obtained with different methods are very different: 21 K [21] and 147 K [20], determined using X‐ray magnetic circular dichroism (XMCD) and magneto‐optical Kerr effect measurements, respectively. Further experimental investigation is necessary to reveal the magnetic and electronic properties of the monolayer FeCl_2_ as well as the interaction between FeCl_2_ and its substrate.

In this study, we report the epitaxial growth of ultrathin magnetic FeCl_2_ films by molecular beam epitaxy (MBE) on a different substrate, Bi(111), which turns out to have a two‐way proximity effect on the electronic and magnetic properties of FeCl_2_/Bi(111) interface (Figure 1a). We grow the FeCl_2_ films on Bi(111) by the same MBE method as we employed to grow FeBr_2_ films [39]. XMCD measurements detect a change from predominantly out‐of‐plane magnetic anisotropy in few‐layer FeCl_2_/Bi(111) to predominantly in‐plane magnetic anisotropy in monolayer FeCl_2_/Bi(111), which is in contrast to the out‐of‐plane easy axis of the monolayer FeCl_2_/Au(111) found in previously reported studies [20, 21]. Angle‐resolved photoelectron spectroscopy (ARPES) measurements reveal an insulating electronic structure, with the valence band top consisting of Fe 3d orbitals located at 2.6 eV below the Fermi level. We find metallic interface states originating from the Bi(111) surface states, which are modified due to charge transfer and backfolding according to the moiré superstructure formed by the FeCl_2_ overlayer. Tight‐binding calculations show that the moiré‐induced interface states form a distinctive trefoil‐shaped Fermi surface.

The reorientation of magnetic anisotropy in monolayer FeCl_2_/Bi(111) demonstrates that substrate proximity can decisively influence the magnetic ground state and interfacial electronic structure of 2D vdW magnets. Although the microscopic mechanism is complex and thus not yet fully understood, this work provides a crucial step toward substrate control of magnetism in 2D vdW materials.

## 2 Results

### 2.1 Structural Characterization of FeCl_2_ Films on Bi(111)

The atomic structure of an FeCl_2_ monolayer is shown in Figure 1b. When deposited on Bi(111), it forms a moiré superstructure of $\left(5\times 5\right)$ FeCl_2_(0001) unit cells on $\left(4\times 4\right)$ Bi(111) unit cells, which was confirmed by low‐energy electron diffraction (LEED) and scanning tunneling microscopy (STM). Figure 1c shows the LEED pattern of the 1.0‐monolayer (ML) FeCl_2_ film on the Bi(111) surface. The spots indicated by the red dotted and blue dashed lines correspond to hexagonal lattices with the lattice constants of 4.49±0.01 Å and 3.60±0.01 Å, which agree with the lattice constants of the (111) plane of the Bi film on the Si(111) substrate (4.48 Å) [40] and the (0001) plane of the bulk FeCl_2_ (3.60 Å) [25, 26], respectively. The other spots, some of which are indicated by the green solid lines, are from the moiré superstructure. In a high‐contrast LEED pattern (Figure S1, Supporting Information), all the LEED spots of the moiré superstructure including higher‐order diffraction are clearly observed and as sharp as the Bi spots, which shows that the superstructure is commensurate with the ${\left(5\times 5\right)}_{{\mathrm{FeCl}}_{2}}$ and ${\left(4\times 4\right)}_{\mathrm{Bi}}$ lattices. No observation of a ring pattern or elongated spots indicates that the monolayer FeCl_2_ film grows epitaxially on the entire Bi(111) surface, where the in‐plane unit vectors of FeCl_2_(0001) are parallel to those of Bi(111). Moiré spots are not observed in the LEED pattern of the monolayer FeCl_2_ grown on the Au(111) surface [21], implying structural modulation of the FeCl_2_ monolayer on Bi(111) dependent on the local geometry with respect to Bi(111) in the moiré unit cell. The pattern of the 2.0‐ML film in Figure 1d shows strong FeCl_2_ spots with weak moiré satellite spots around each of them. The thickness‐dependent change in the lattice constant of FeCl_2_ was negligible. The sixfold FeCl_2_ pattern indicates that two FeCl_2_ domains with orientations rotated by 180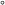 with respect to each other coexist on the threefold Bi(111) surface.

A large‐scale STM topographic image of the 1.0‐ML FeCl_2_ film (Figure 1e) shows that most of the area is covered with monolayer FeCl_2_. The morphology is similar to that reported for the pristine Bi(111) film [41], indicating that the monolayer FeCl_2_ grows without breaking the original terrace and step structure. Darker areas on a terrace, one of which is denoted by the white arrow in the middle part, are bare Bi(111). Near the step edges, small islands of bilayer FeCl_2_ are observed both on upper and lower terraces. The area of the bare Bi(111) and the bilayer FeCl_2_ are approximately 10% and 1%, respectively. The atomically resolved STM image and the fast Fourier transform (FFT) pattern of the monolayer (Figure 1g,h) exhibit a hexagonal lattice with a periodicity of a=b=3.5±0.4 Å, which is consistent with the FeCl_2_ lattice constant derived from the LEED pattern. The moiré superstructure is clearly seen as the intensity modulation with a periodicity of 18.2±0.3 Å (the green solid lines). The LEED and STM results of the 1.0‐ML film demonstrate that the monolayer FeCl_2_ film epitaxially grows with a large domain size of more than 100 nm. The 2.0‐ML FeCl_2_ film (Figure 1f) has more complex structure consisting not only of bilayer but also of monolayer and trilayer. The bilayer FeCl_2_ covers only 80% of the surface and the monolayer covers 12%, which is observed as dark depressions enclosed by the bilayer. Although the bilayer formation is not yet completed, the trilayer (8%) already starts to grow from the step edges of the bilayer mainly toward lower terraces. This complex structure of the 2.0‐ML film suggests that the FeCl_2_ film does not follow a perfect layer‐by‐layer growth mode after the formation of the monolayer film on the Bi(111) surface.

### 2.2 Magnetic Anisotropy of Mono‐ and Few‐Layer FeCl_2_/Bi(111)

**FIGURE 2:**
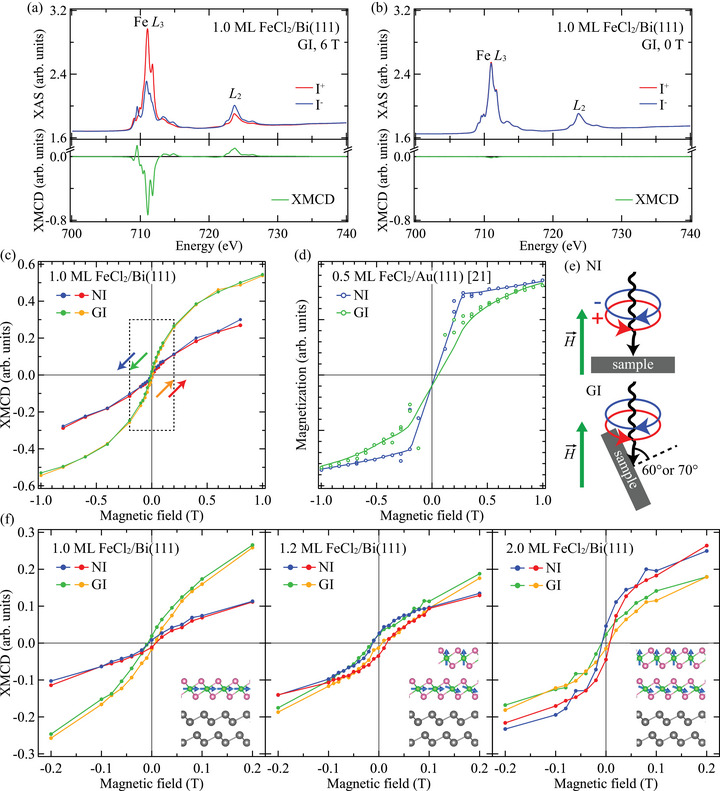
X‐ray absorption spectra measured with (red) circular right and (blue) circular left polarizations and (green) XMCD spectra at the Fe $L_{2,3}$ edges of the 1.0‐ML FeCl_2_ film on Bi(111) acquired in the GI geometry at 2 K with magnetic fields of (a) 6 T and (b) 0 T. (c) XMCD magnetization curves obtained for the Fe $L_{3}$ edge of the 1.0‐ML FeCl_2_ film on Bi(111). The dotted rectangle denotes the region shown in (f). (d) XMCD magnetization curves of the 0.5‐ML FeCl_2_ film on Au(111) reproduced from Ref. [21]. (e) Orientation of the NI and GI geometries. (f) Thickness dependence of the magnetization curves of the FeCl_2_ films on Bi(111). Schematic illustrations of the magnetic order of the 1.0‐ML, 1.2‐ML and 2.0‐ML FeCl_2_ films are displayed on the bottom right. The data of the 1.2‐ML FeCl_2_ film were taken at 7 K at the BOREAS beamline, and those of the 1.0‐ML and 2.0‐ML films were obtained at 2 K at the DEIMOS beamline.

**FIGURE 3:**
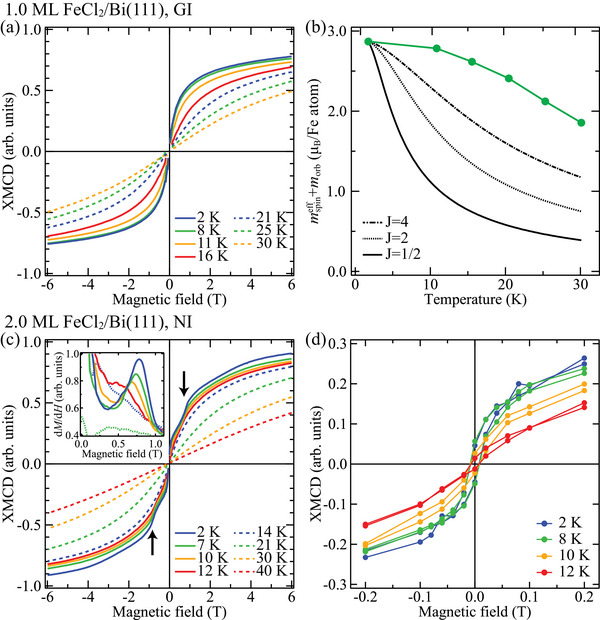
Temperature dependence of XMCD magnetization curves of (a,b) the 1.0‐ML FeCl_2_ film in the GI geometry and (c,d) the 2.0‐ML FeCl_2_ film in the NI geometry. (a,c) Magnetization curves obtained for the Fe $L_{3}$ edge. First derivatives of the magnetization curves in (c) are shown in the inset of (c). M and H represent the XMCD intensity and magnetic field, respectively. (b) Magnetic moment evaluated by the sum‐rule analysis as a function of temperature. The black curves represent the Brillouin functions for J=1/2 (solid), 2 (dotted), and 4 (dashed‐dotted) expected for paramagnetic states. (d) Hysteresis loops obtained from XMCD spectra at each magnetic field. All the spectra were obtained at the DEIMOS beamline. In (a) and (c), artificial spikes around 0 T typical for the total electron yield (TEY) detection mode were removed.

We investigated the magnetic properties of the ultrathin FeCl_2_ films by X‐ray absorption spectroscopy (XAS) and XMCD measurements. Figure 2a,b shows the X‐ray absorption spectra measured with right and left circularly polarized light and XMCD spectra of the 1.0‐ML FeCl_2_ films in the grazing‐incidence (GI) geometry with applied magnetic fields of 6 T and 0 T, respectively. The Fe $L_{2,3}$ XAS lineshapes at 0 T agree with those measured for samples with the high‐spin ${\mathrm{Fe}}^{2+}$ state including bulk FeO and few‐monolayer FeBr_2_ films [18, 42, 43], indicative of the growth of the single‐phase FeCl_2_ films from the monolayer. A large XMCD signal of 82% at the Fe $L_{3}$ edge with respect to the XAS edge jump averaged for the two polarizations was observed at a magnetic field of 6 T, while a very small XMCD signal (1.7%) was detected at 0 T. In Figure 2c, field‐dependent XMCD curves of the 1.0‐ML FeCl_2_ film on Bi(111) in the normal‐incidence (NI) and GI geometries are compared. Whereas the NI curve is nearly linear, the GI curve has an S‐shape with a much steeper slope near 0 T than the NI one, indicating that the monolayer FeCl_2_ on Bi(111) has a magnetic order with a predominantly in‐plane easy axis. The presence of the magnetic order is corroborated by the temperature dependence of the magnetic moment which does not follow a Brillouin function (see Figure 3b). Considering that the out‐of‐plane easy axis of the unit layer in the bulk FeCl_2_ [11, 25] is preserved in the monolayer FeCl_2_ on Au(111) [20, 21] (see Figure 2d), it is clear that the Bi(111) substrate has a strong effect on the magnetic anisotropy of the overgrown FeCl_2_ monolayer, reorienting the easy axis into the surface plane.

Figure 2f displays the thickness dependence of the magnetization curves in the magnetic field range of ±0.2 T. The XMCD intensities of the 1.0‐ML film have a small curvature both in the NI and GI geometries, which is steeper for GI. Small hysteresis and remanent magnetization are detected both in the NI and GI geometries, which is consistent with the very small XMCD signal at 0 T in Figure 2b. When increasing the thickness to 1.2 ML and 2.0 ML, both remanence and hysteresis loop opening evolve in the NI curves, whereas the GI curves remain narrow. The remanence and hysteresis loop opening in the NI geometry are negligibly small in the 1.0‐ML film, but appear very clearly in the 1.2‐ML film. The 1.2‐ML film is likely composed of the monolayer and bilayer with minimal contribution from the trilayer, which typically grows from the step edges of the bilayer islands. This observation shows that the remanence and hysteresis loop opening are attributed to the bilayer FeCl_2_, rather than to the monolayer or trilayer. This thickness dependence shows that only the monolayer FeCl_2_ on Bi(111) exhibits the in‐plane anisotropy and that the out‐of‐plane anisotropy of the bulk FeCl_2_ is recovered on the transition to the bilayer, demonstrating that the effect of the Bi(111) substrate is restricted to the FeCl_2_ layer directly facing Bi(111). The schematic images in the lower‐right part of the panels in Figure 2f illustrate the magnetic configurations of the monolayer and bilayer FeCl_2_ on Bi(111). For the bilayer, the schematic represents one possible configuration, and other configurations cannot be excluded, such as interlayer ferromagnetic coupling. Nevertheless, given the presence of the remanent magnetization, the bilayer is not expected to have a simple interlayer antiferromagnetic configuration of bulk FeCl_2_, which would result in zero remanence. Our experimental results provide strong evidence that the non‐magnetic Bi(111) substrate plays a key role in modifying the magnetic anisotropy of the 2D vdW magnet at the monolayer and bilayer limit.

The magnetic moments were evaluated using the sum‐rule analysis [44] for the XMCD spectra at 6 T and 2 K. The obtained values of the effective spin magnetic moment $m_{\mathrm{spin}}^{\mathrm{eff}}\left(=m_{s}+7m_{T}\right)$ and the orbital magnetic moment $m_{\mathrm{orb}}$ are summarized in Table 1. Here, $m_{s}$ is the spin magnetic moment and $m_{T}$ is the magnetic dipole moment. We used four as the number of Fe 3d holes when calculating the moments. The total magnetic moments of the 1.0‐ML and 2.0‐ML films (2.7 – 2.9 $\mu_{B}$) are almost the same and are lower than 4.3 $\mu_{B}$ of the bulk FeCl_2_ [25, 27]. The orbital moments of 0.59 – 0.66 $\mu_{B}$ are consistent with 0.71 and 0.59 $\mu_{B}$ theoretically calculated for the bulk and monolayer FeCl_2_, respectively [36, 45], whereas the spin moments of 2.09 – 2.22 $\mu_{B}$ are smaller than 3.42 and 3.56 $\mu_{B}$ calculated for the bulk and monolayer FeCl_2_, respectively. Such small spin moments were also reported in the XMCD studies on the monolayer FeBr_2_ and FeCl_2_ on Au(111) [18, 21]. This is because the magnetization curve is not yet saturated even at the maximum available field of 6 T (see Figure 3a,c).

**TABLE 1:** (left) Magnetic moments in $\mu_{B}$ per Fe atom evaluated using the sum rule analysis for the XMCD spectra obtained at 6 T and 2 K for the 1.0‐ML and 2.0‐ML FeCl_2_ films on Bi(111) in the GI and NI geometries. (right) Calculated magnetocrystalline anisotropy energy ($E_{\mathrm{MCA}}$), magnetic shape anisotropy energy ($E_{\mathrm{MSA}}$) and total magnetic anisotropy energy ($E_{\mathrm{MAE}}$) in meV per Fe atom.

	GI		NI				
	$m_{\mathrm{spin}}^{\mathrm{eff}}$	$m_{\mathrm{orb}}$	$m_{\mathrm{spin}}^{\mathrm{eff}}$	$m_{\mathrm{orb}}$	$E_{\mathrm{MCA}}$	$E_{\mathrm{MSA}}$	$E_{\mathrm{MAE}}$
1.0 ML	2.09±0.05	0.60±0.02	2.18±0.05	0.59±0.01	−0.07±0.13	−0.07±0.00	−0.14±0.13
2.0 ML	2.17±0.06	0.63±0.02	2.22±0.06	0.66±0.02	0.30±0.07	−0.08±0.00	0.22±0.07

In order to get a quantitative insight into the anisotropy change between the monolayer and bilayer FeCl_2_ on Bi(111), we calculate the magnetic anisotropy energy ($E_{\mathrm{MAE}}$), which is given by $E_{\mathrm{MAE}}=E_{\mathrm{MCA}}+E_{\mathrm{MSA}}$. Here, $E_{\mathrm{MCA}}$ is the magnetocrystalline anisotropy energy and $E_{\mathrm{MSA}}$ is the magnetic shape anisotropy energy. The values of $E_{\mathrm{MCA}}$, $E_{\mathrm{MSA}}$ and $E_{\mathrm{MAE}}$ obtained for the 1.0‐ML and 2.0‐ML FeCl_2_ films are presented in Table 1 (see Supporting Information [46] for the detailed evaluation procedure of the anisotropy energies). The magnetic shape anisotropy energy $E_{\mathrm{MSA}}$ originating from dipole‐dipole interaction is calculated to be −0.07 and −0.08 meV per Fe atom for the monolayer and bilayer FeCl_2_, respectively. The magnetocrystalline anisotropy energy $E_{\mathrm{MCA}}$ is evaluated from the orbital‐moment anisotropy to be −0.07 meV for 1.0 ML and 0.30 meV for 2.0 ML. Therefore, the total magnetic anisotropy energy $E_{\mathrm{MAE}}$ is −0.14 meV for the 1.0‐ML film and 0.22 meV for the 2.0‐ML film. The reversal of the sign of the $E_{\mathrm{MAE}}$ value is consistent with the anisotropy change observed in the magnetization curves in Figure 2f corresponding to the in‐plane and out‐of‐plane easy axes for the monolayer and bilayer, respectively. The absolute values of $E_{\mathrm{MAE}}$ are comparable to 0.13 meV reported for the monolayer FeCl_2_ on Au(111) [21]. The monolayer FeCl_2_ on Bi(111) exhibits a larger magnitude of in‐plane anisotropy than monolayer NiCl_2_ ($E_{\mathrm{MAE}}=-0.07$ meV [21]) and monolayer CrCl_3_ ($E_{\mathrm{MAE}}=-0.09$ ‐ −0.11 meV [47]), both of which possess an in‐plane easy axis in their bulk crystals. Note that there are other contributions to the magnetic anisotropy such as magnetoelastic anisotropy [48] and exchange anisotropy [47, 49]. However, considering that the FeCl_2_ films on Bi(111) have almost the same lattice constant as the bulk FeCl_2_ and that they have a band gap larger than 2.5 eV (see ARPES measurements below), these contributions are expected to be much smaller than the magnetocrystalline and magnetic shape anisotropies.

### 2.3 Temperature Dependence of Magnetic Properties of FeCl_2_

We discuss the temperature dependence of the magnetic properties of the ultrathin FeCl_2_ films. Figure 3a shows the magnetization curves of the 1.0‐ML FeCl_2_ film on Bi(111) in the GI geometry at different temperatures from 2 to 30 K. The S‐shaped curve becomes gradually flatter as the temperature increases above 8 K. The temperature dependence of the total magnetic moment at 6 T obtained by the sum‐rule analysis (Figure 3b) does not follow a quick decay of the Brillouin functions predicted for the paramagnetic states as illustrated by the black curves for J=1/2, 2 and 4, where J=2 is approximately the case for FeCl_2_. This temperature dependence supports the existence of long‐range order in the monolayer FeCl_2_.

Modified Arrott‐plot analysis is used to determine the critical exponents β and γ and the resulting magnetic universality class by fitting $M^{1/\beta }$ vs. ${\left(H/M\right)}^{1/\gamma }$, producing a set of parallel straight lines with one at $T_{c}$ passing the origin [5, 47, 50]. Here, M and H correspond to the XMCD intensity and magnetic field, respectively. We have examined eight 2D and 3D models including mean‐field, Ising, XY and Heisenberg models, but all the examined models fail to produce a set of straight lines for the data from 2 to 30 K (see Figure S3, Supporting Information). This finding should motivate future studies to determine the magnetic universality as well as the transition temperature of the monolayer FeCl_2_ on Bi(111).

Figure 3c,d shows the magnetization curves of the 2.0‐ML FeCl_2_ film on Bi(111) in the NI geometry at temperatures from 2 to 40 K. We first focus on the hysteresis loop opening of the bilayer FeCl_2_ (Figure 3d), which is observed as a large jump near 0 T in the wide field range scans (±6 T) (Figure 3c). With increasing temperature, the hysteresis loop opening becomes smaller and almost disappears at 12 K, suggesting the onset of the ferro‐ to paramagnetic transition. Another characteristic feature in the NI magnetization curve of the 2.0‐ML film is an abrupt change of curvature around ±0.8 T indicated by the black arrows in Figure 3c, which yields a peak in the first derivative curve as shown in the inset of Figure 3c. Given that the 2.0‐ML film consists not only of the bilayer but also of the trilayer (see Figure 1f), this curvature change at ±0.8 T is likely attributed to a spin‐flop transition of the trilayer FeCl_2_ having an AFM ground state with the out‐of‐plane easy axis. The transition field of 0.8 T is smaller than that of the metamagnetic transition of the bulk FeCl_2_ (∼1.5 T) [27, 28], suggesting a weaker interlayer interaction in the trilayer film than that of the bulk. As the temperature increases, the abrupt curvature change becomes less clear and the transition field gradually shifts from 0.8 T toward 0 T, and then it disappears at 14 K, as clearly found in the first derivative curves in the inset of Figure 3c. This particular behavior suggests the magnetic transition temperatures of the bilayer and trilayer FeCl_2_ are around 12 K and 14 K, respectively. These values are about half of the transition temperature of the bulk FeCl_2_ (24 K).

### 2.4 Electronic Structure of FeCl_2_

**FIGURE 4:**
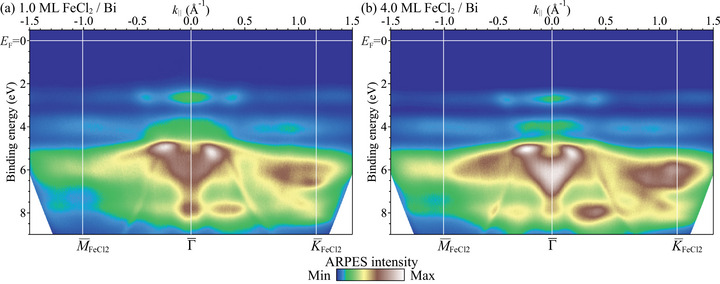
Valence band structure of the (a) 1.0‐ML and (b) 4.0‐ML FeCl_2_ films on Bi(111) at room temperature along $\bar{M}-\bar{\Gamma }-\bar{K}$ of the FeCl_2_ SBZ measured with He Iα.

**FIGURE 5:**
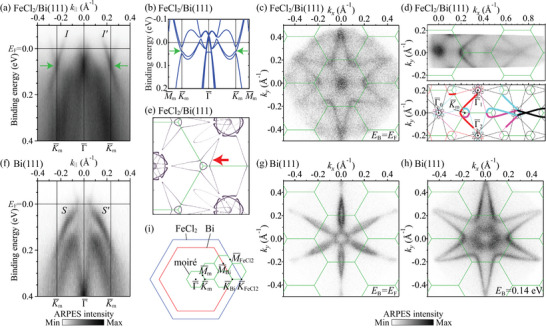
(a) ARPES band dispersion along ${\bar{K}}_{m}-\bar{\Gamma }-{\bar{K}}_{m}$ of the 1.0‐ML FeCl_2_ film on Bi(111). (b) Band dispersion along ${\bar{M}}_{m}-{\bar{K}}_{m}-\bar{\Gamma }-{\bar{K}}_{m}-{\bar{M}}_{m}$ of the moiré superstructure calculated by the tight‐binding method. (c) Fermi surface map and (d) high‐statistics Fermi surface map of the 1.0‐ML FeCl_2_ film on Bi(111). The Fermi surface is schematically illustrated in the bottom panel of (d). Stars centered at the ${\bar{\Gamma }}_{m}$ points are represented by thin curves and the parts strongly observed by ARPES are emphasized by thick curves. (e) Calculated Fermi surface of the moiré superstructure. The Fermi level in the calculation was determined from the qualitative agreement of the Fermi surface shape between the experiment and calculation. (f) ARPES band dispersion, and constant energy maps at (g) $E_{B}$ = $E_{F}$ and (h) $E_{B}$ = 0.14 eV of Bi(111). (i) SBZs of the (blue) FeCl_2_(0001), (red) Bi(111), and (green) moiré lattices. The energy window of the constant energy maps in (c, d, g, h) is 20 meV. The green solid lines in (c, d, e, g, h) represent the moiré mini‐SBZs. The green arrows in (a, b) indicate the crossing point of the moiré‐induced interface states. The red arrow in (e) indicates the trefoil Fermi surface at the ${\bar{K}}_{m}$ point. The ARPES data were obtained with He Iα (hν=21.2 eV) at room temperature.

To determine the electronic structure of FeCl_2_/Bi(111), we performed ARPES measurements and compared the results to the electronic structure of the bare Bi(111) surface. Figure 4 presents the ARPES valence band structure of the 1.0‐ML and 4.0‐ML FeCl_2_ films on Bi(111) at room temperature along $\bar{M}-\bar{\Gamma }-\bar{K}$ of the FeCl_2_ surface Brillouin zone (SBZ) (see Figure 5i). The FeCl_2_ films have similar band features to those of the monolayer and few‐layer FeBr_2_ films on Bi(111), which have two nearly flat bands mainly composed of localized Fe 3d orbitals at binding energies of 2.2 and 3.2 eV and dispersive bands below 3.7 eV with higher Br 4p contributions [39]. From the comparison of the band structure between FeBr_2_ and FeCl_2_, for the 1.0‐ML FeCl_2_ film (Figure 4a), the two nearly flat bands at 2.6 and 4.1 eV are considered to be derived mainly from the Fe 3d orbitals and the dispersive bands below 4 eV are to have larger contributions from Cl 3p orbitals. The 4.0‐ML film exhibits almost the same band dispersions as those of the 1.0‐ML film except a shift of ∼0.1 eV toward higher binding energy, sharpening of the Fe 3d flat bands, and broadening of the Cl‐derived bands at around 6 eV at $\bar{\Gamma }$, which corresponds to the appearance of the Cl–Cl interaction between the neighboring layers. No FeCl_2_‐derived electronic states are observed near the Fermi level, showing that the monolayer FeCl_2_ is insulating and that the valence band maximum is located at 2.6 eV below the Fermi level. The insulating property of the bulk FeCl_2_ remains down to the monolayer as is the case with FeBr_2_ [39]. The observed insulating property agrees with the scanning tunneling spectroscopy studies on the monolayer FeCl_2_ film on graphite and Au(111) substrates [12, 13, 20, 21], and an ARPES study on the monolayer FeCl_2_ on Bi_2_Se_3_ [51], demonstrating that the insulating nature of the monolayer FeCl_2_ is not an extrinsic property induced by the substrates, but an intrinsic one.

### 2.5 Metallic Interface States

We found metallic states in the bandgap of the 1.0‐ML FeCl_2_ film on Bi(111) (Figure 5a), which originate from the metallic Bi surface states as discussed below. The states consist of sharp Dirac‐cone‐like crossing bands and a blurred feature at $\bar{\Gamma }$ near the Fermi level with broader bands dispersing downward toward $\bar{K}$. Here, we focus on analysing the origin of the Dirac‐cone‐like bands. There are no such crossing bands in the band structure of the pristine Bi(111) surface, as shown in Figure 5f. The stronger branches I and $I^{\prime }$ of the crossing bands form a six‐pointed star in the Fermi surface map in Figure 5c. This Fermi surface is different from that of the pristine Bi(111) surface in Figure 5g composed mainly of a hexagonal electron pocket at $\bar{\Gamma }$ and six teardrop‐shaped hole pockets along $\bar{\Gamma }$–$\bar{M}$. Interestingly, a similar star shape is found in the constant energy map of the Bi(111) surface at a binding energy of around 100 meV (Figure 5h), which is formed by the spin‐polarized surface states S and $S^{\prime }$ (Figure 5f) [52, 53]. The values of the wave vectors of the I and $I^{\prime }$ bands at the Fermi level along $\bar{\Gamma }$–$\bar{K}$ coincide with those of the Bi(111) surface states S and $S^{\prime }$ bands at a binding energy of 0.14 eV. These results suggest that adding monolayer FeCl_2_ to the Bi(111) surface induces an upward band shift of ∼0.14 eV via a charge transfer from Bi to FeCl_2_. The amount of the transferred charge was determined to be 0.039±0.002 electrons per FeCl_2_ unit cell from the change in the Fermi surface Luttinger area.

The crossing points of the bands indicated by the green arrows in Figure 5a are located at the $\bar{K}$ point of the moiré mini‐SBZ (the ${\bar{K}}_{m}$ point). In order to clarify the origin of the crossing bands, we measured the high‐statistics Fermi surface map of the 1.0‐ML FeCl_2_ film on Bi(111) (the top panel of Figure 5d).

The crossing bands form a nearly circular Fermi contour at the ${\bar{K}}_{m}$ point. This circular contour is derived from the overlapping of the inner corners of the three stars centered at the ${\bar{\Gamma }}_{0}$, ${\bar{\Gamma }}_{1}$ and ${\bar{\Gamma }}_{2}$ points as schematically drawn in the bottom panel of Figure 5d, where the star is replicated according to the moiré mini‐SBZs. The bands strongly observed by ARPES are drawn with thick curves. The other contours in the ARPES data can also be explained by the backfolding of the star Fermi contour according to the moiré mini‐SBZs. The small circle at the ${\bar{\Gamma }}_{0}$ point in the experimental Fermi surface is likely attributed to the overlapping of the outer corners of the stars centered at the ${\bar{\Gamma }}_{m}$ points of the six moiré mini‐SBZs surrounding the first mini‐SBZ. We confirmed that the observed backfolding is an intrinsic feature of the initial states, rather than the umklapp‐scattering effect during the photoemission process [54, 55] by measuring the Fermi surface maps and band dispersions with different polarizations of the incident light (see Figure S4, Supporting Information). The ARPES results indicate that the metallic states of the monolayer FeCl_2_ on Bi(111) are the interface states originating from the Bi(111) surface states modified by the hole doping and the backfolding according to the SBZs of the moiré superstructure formed by the FeCl_2_ overlayer. Since the conical bands are derived from the spin‐polarized bands of the Bi(111) surface state, they are expected to be spin‐polarized. In addition, the interface states will play a significant role in the transport properties of the FeCl_2_/Bi(111) heterostructure, given that the Bi surface states dominantly contribute to the transport properties of pristine Bi films thinner than ∼8 nm [56, 57].

The I and $I^{\prime }$ bands in Figure 5a show almost linear dispersion. However, ARPES measurement at 35 K, which is still higher than the magnetic transition temperature of the bulk FeCl_2_ (24 K), revealed that the bands exhibit an anticrossing behavior at the ${\bar{K}}_{m}$ point and the gap is estimated to be ∼30 meV (see Figure S5, Supporting Information). The opening of a hybridization gap between the original and the backfolded band at the mini‐SBZ boundary further proves that the replica bands are due to the intrinsic moiré potential acting on the initial states in the photoemission process. The observation of the moiré‐potential‐induced replica bands and the anticrossing gap suggests a strong interaction between FeCl_2_ and Bi(111), which is expected to modify the magnetic behavior of the monolayer FeCl_2_. This is a stark contrast to the weak electronic interaction between the monolayer FeCl_2_ and the Au(111) surface, where the unperturbed herringbone reconstruction was observed by STM even after the monolayer FeCl_2_ formation and the out‐of‐plane magnetic anisotropy remains [20, 21].

### 2.6 Trefoil Fermi Surface and Lifshitz Transition

We performed a tight‐binding calculation to simulate the effect of the moiré potential on the Bi surface states. Figure 5b,e shows the calculated band dispersion and Fermi surface, respectively. The details of the calculation are described in the Supporting Information [46]. The calculated results reproduce well the crossing bands and the Fermi contours near the ${\bar{K}}_{m}$ point. At the ${\bar{K}}_{m}$ point, three bands corresponding to the star Fermi contours centered at the three neighboring ${\bar{\Gamma }}_{m}$ points are crossed and hybridized to open a small gap. The moiré coupling strength in the calculation was chosen so that the calculated gap size (27 meV) at ${\bar{K}}_{m}$ is consistent with the experimental one (∼30 meV).

We found that the Fermi surface of the FeCl_2_/Bi(111) heterostructure displays a threefold (“trefoil”) symmetry at the corners (the ${\bar{K}}_{m}$ points) of the hexagonal mini‐SBZ.

With our moiré band model, we revealed that the center of the trefoil is detached from the outliers (the“leaves”) due to the moiré potential, creating a small isolated Fermi pocket in the middle, as shown in Figure 5e.

The trefoil Fermi surface is a motive enabled when the hybridization gap between the folded bands due to the moiré potential is not too large. A large hybridization gap would induce a strong distortion from the original band dispersions, which can break the trefoil feature. As discussed in Section 8.2.3 in the supporting information [46], the moiré‐induced band hybridization at ${\bar{K}}_{m}$ is given by the product of the moiré coupling strength and the expectation value of the $C_{3z}$ rotation operator of the Bloch states. As the Bloch states near ${\bar{K}}_{m}$ are a superposition of states with different $C_{3z}$ eigenvalues, this expectation value is very small (see Figure S7e, Supporting Information). This small $C_{3z}$ expectation value leads to a suppression of the hybridization gap down to about 30 meV – providing a symmetry reason for why the trefoil Fermi surface is observable.

The Fermi surface changes from entangled trefoils to disentangled branches as the Fermi level intersects different parts of the bands around ${\bar{K}}_{m}$ (see Figure S8e,f). Although this transition does not break any conventional symmetry, it alters the connectivity of the Fermi surface in a fundamental way.

The small splitting of the Fermi pocket at the center of the trefoil can fall below the ARPES energy and momentum resolution. One way to experimentally detect the small pocket and inter‐pocket tunneling is through magnetic breakdown and Fermi‐surface reconstruction, where electrons can tunnel between nearby cyclotron orbits in a sufficiently strong magnetic field. Under a strong magnetic field, the central small pocket can connect to the outliers, forming a multiply connected trajectory that features multiple quantum‐oscillation frequencies, directly revealing the presence of distinct sub‐bands. In a previous study, ARPES measurements on the Au(111) surface with the $\left(22\times \sqrt{3}\right)$ herringbone reconstruction revealed a three‐lobed Fermi contour that winds around the SBZ like a trefoil knot [58]. This Fermi contour is similar to what we have observed for the FeCl_2_/Bi(111) heterostructure. However, the trefoil Fermi surface of Au(111) is formed by the superimposed photoemission signal of three independent domains of the reconstruction. Therefore, no magnetic breakdown is expected in that case. Here, our monolayer FeCl_2_/Bi(111) interface provides a compelling platform to observe this exotic fermiology and the associated magnetic‐breakdown phenomena. Our theoretical scheme introduced in the Methods section and the supporting information [46] together with our code (see the code availability) can be readily applied to study other moiré heterostructures.

## 3 Discussion

Let us compare our experimental results and previous DFT calculations to discuss the possible origin of the reorientation of the magnetic easy axis observed in the monolayer FeCl_2_ on Bi(111).

Our ARPES and XMCD experiments demonstrate that the monolayer FeCl_2_ has the insulating electronic structure and the predominantly in‐plane magnetic anisotropy. The electronic states around the Fermi level and the magnetic properties are mainly attributed to the Fe 3d electrons [34, 35, 36, 38]. The octahedrally coordinated ${\mathrm{Fe}}^{2+}$ ion has six 3d electrons with the configuration of $t_{2g}^{3↑}e_{g}^{2↑}$ and $t_{2g}^{1↓}$. The down‐spin $t_{2g}$ orbitals compose the valence band maximum of the monolayer FeCl_2_. The trigonal distortion of the octahedra along the [0001] direction, SOC and electron correlation effect further split the $t_{2g}$ triplet into $a_{1g}$, $e_{g+}^{\prime }$ and $e_{g-}^{\prime }$ singlets with $l_{z}=0,+1,-1$, respectively. The electronic structure of the monolayer FeCl_2_ is calculated to be half‐metallic when the splitting of the singlets is small, but it becomes insulating when the splitting is large [35], which agrees with our ARPES result. Therefore, monolayer FeCl_2_ can be understood as a spin‐orbit‐assisted Mott insulator. In the calculations showing the insulating electronic structure, either $a_{1g}$ or $e_{g+}^{\prime }$ state is the ground state, depending on the strength of the SOC and electron correlation effect [35]. The $e_{g+}^{\prime }$ state has the out‐of‐plane magnetic anisotropy with a sizeable orbital moment of 0.59 $\mu_{B}$, whereas the $a_{1g}$ state has the in‐plane anisotropy with a smaller orbital moment of 0.23 $\mu_{B}$ [36]. The value of the orbital moment calculated for the $e_{g+}^{\prime }$ state shows a good agreement with those of 0.59 and 0.66 experimentally obtained for the 1.0‐ML and 2.0‐ML FeCl_2_ films (Table 1), suggesting that the $e_{g+}^{\prime }$ state is the ground state of the monolayer and bilayer FeCl_2_.

For the monolayer FeCl_2_ on Bi(111), we speculate that the proximity effect from the Bi(111) surface could affect the relative energy levels of the FeCl_2_ monolayer, resulting in a larger contribution of the $a_{1g}$ state to the ground state.

The different contributions of the Fe 3d orbitals to the ground state may be related to the broadening of the flat band at 2.6 eV observed in the 1.0‐ML FeCl_2_ film compared to the 4.0‐ML film (Figure 4). This broadening is unlikely to originate from structural disorder since our STM images (Figure 1e,f) demonstrate that the 1.0‐ML sample has higher crystallinity than multilayer samples. A previous DFT work [35] shows that stronger SOC stabilizes the $e_{g+}^{\prime }$ ground state with the out‐of‐plane easy axis. Charge transfer is another factor influencing the magnetic anisotropy; electron doping in monolayer FeCl_2_ was predicted to enhance the out‐of‐plane anisotropy [59]. Given that Bi has a strong SOC and charge transfer from Bi to FeCl_2_ was observed by ARPES, the above two factors intuitively enhance the out‐of‐plane anisotropy, which is contrary to our results. Lattice strain is another factor affecting the energy levels and magnetic properties of FeCl_2_; tensile strain is predicted to weaken the out‐of‐plane magnetic anisotropy by DFT calculations [36, 60]. An effective lattice strain on the monolayer FeCl_2_ on Bi(111) may arise from the structural modulation induced by the moiré potential. Our LEED patterns (Figure 1c; Figure S1, Supporting Information) reveal pronounced moiré spots and the STM image (Figure 1g) shows apparent height modulation as large as ∼1 Å, which is comparable to the corrugation of monolayer graphene strongly bonded to a Ru(0001) substrate [61, 62], implying that the structure of the FeCl_2_ monolayer is modulated dependent on the local geometry with respect to Bi(111), even though the averaged in‐plane lattice constant agrees with that of bulk FeCl_2_. In addition to the aforementioned effects, other factors, such as the moiré‐potential‐induced spin‐polarized interface states, can also affect the magnetic anisotropy. The combination of these effects and the contribution of magnetic shape anisotropy is expected to realize the predominantly in‐plane magnetic anisotropy of the monolayer FeCl_2_ on Bi(111). These results motivate future studies into the microscopic atomic structure and electronic states, and detailed magnetic structure of the monolayer FeCl_2_/Bi(111), as well as theoretical modelling of the heterostructure, to reveal the origin of the magnetic anisotropy reorientation.

## 4 Conclusion

We successfully grew the epitaxial monolayer and few‐layer FeCl_2_ films on the Bi(111) surface by MBE. LEED and STM showed that the monolayer FeCl_2_ has a moiré superstructure of $\left(5\times 5\right)$ FeCl_2_(0001) unit cells on $\left(4\times 4\right)$ Bi(111) unit cells. XMCD measurements revealed that the monolayer FeCl_2_ on Bi(111) has the magnetic order with the predominantly in‐plane easy axis, which differs from the out‐of‐plane magnetic anisotropy of the bulk FeCl_2_ and the monolayer FeCl_2_ that is only weakly coupled to the Au(111) substrate. ARPES demonstrated that the monolayer FeCl_2_ on Bi(111) has the insulating electronic structure with the valence band top at 2.6 eV below the Fermi level consisting of the Fe 3d orbitals. In the band gap of the FeCl_2_, metallic interface states were found. The interface states originate from the Bi(111) surface states modified by the charge transfer and the backfolding according to the moiré mini‐SBZ induced by the FeCl_2_ overlayer, suggesting the strong interaction between FeCl_2_ and Bi(111). Tight‐binding calculation showed that the interface states form a unique trefoil‐shaped Fermi surface. Our work demonstrates a substrate‐induced control of magnetic anisotropy in a monolayer magnet, crucial to design spintronic devices based on 2D vdW magnets and their heterostructures.

## 5 Methods

### 5.1 FeCl_2_ Thin‐film Growth

Ultrathin FeCl_2_ films were grown on single‐crystalline Bi(111) films with 6‐nm thickness on Si(111) substrates in an ultrahigh vacuum (UHV) chamber with a base pressure of $2\times 10^{-10}$ mbar. The Si(111) substrates (n‐type) were cleaned by direct‐current heating at 1500 K to obtain sharp $\left(7\times 7\right)$ LEED spots. Bi (99.999%, Sigma Aldrich) was deposited on the clean Si(111) $\left(7\times 7\right)$ surface at room temperature and then the Bi films were annealed at around 400 K. FeCl_2_ anhydrous beads (99.99%, Sigma Aldrich) in a tantalum crucible were heated at 570 K and deposited on the Bi(111) surface at room temperature followed by annealing at 490 K. The deposition rate of FeCl_2_ was calibrated by observing the LEED patterns; one monolayer (ML) corresponds to the strongest intensity of the moiré spots. The FeCl_2_ films were grown in the MBE chamber at the Max Planck Institute of Microstructure Physics and transferred to each chamber for ARPES, STM and XMCD with a base pressure lower than $1\times 10^{-9}$ mbar using a home‐built sample transfer system, which enables us to transfer the samples under UHV or inert gas atmosphere. The preservation of the sample quality after the transfers was confirmed by LEED. Identical moiré LEED pattern was observed in three different monolayer FeCl_2_/Bi(111) samples separately grown for STM, XMCD and ARPES measurements as shown in Figure S2 (Supporting Information) [46].

### 5.2 XMCD Measurements

The XAS and XMCD experiments were carried out at the DEIMOS beamline of the SOLEIL synchrotron and the BOREAS beamline [63] of the ALBA synchrotron. The X‐ray absorption spectra at the Fe $L_{2,3}$ edges were recorded by a total electron yield (TEY) mode in the normal‐incidence (NI) geometry (the X‐ray beam is perpendicular to the sample surface) and the grazing‐incidence (GI) geometry (the beam is tilted by $60^{\circ }$ or $70^{\circ }$ from the surface normal) with a magnetic field up to 6 T applied along the beam direction. The orientations of the NI and GI geometries are schematically drawn in Figure 2e. The NI and GI geometries were used to measure the magnetic properties in the out‐of‐plane and in‐plane directions, respectively. Magnetic‐field dependence of the XMCD intensities in Figure 2c,f was extracted from the XMCD spectra normalized to the Fe $L_{3}$ XAS edge jump at each magnetic field to avoid artifacts near the zero magnetic field typical for the TEY detection mode.

### 5.3 ARPES Measurements

ARPES experiments were performed using a SPECS Phoibos 225 hemispherical electron energy analyzer with monochromatized He Iα radiation (hν=21.2 eV) at the Max Planck Institute of Microstructure Physics and a SPECS Phoibos 150 analyzer with linearly polarized synchrotron radiation (hν=30 eV) at the Bloch beamline of the MAX IV Laboratory.

### 5.4 STM Measurements

STM measurements (Omicron VT‐STM) were performed at room temperature in the constant‐current mode with a mechanically sharpened Pt/Ir alloy tip. The tip was calibrated on the surface of a Au(111) single crystal before scanning.

### 5.5 Statistical Analysis

Data are presented as the mean ± standard deviation for quantitative measurements. The in‐plane lattice constants were determined using five independent LEED patterns and three independent STM images. The magnetic moments shown in Table 1 were obtained from three and two independent XMCD spectra at each geometry for 1.0‐ML and 2.0‐ML FeCl_2_ samples, respectively. The transferred charge was obtained by evaluating the sizes of the two independently measured Fermi surface maps both for monolayer FeCl_2_/Bi(111) and pristine Bi(111). All data processing and statistical analysis were performed using Igor Pro.

### 5.6 Calculation

First‐principles calculation on the Bi band structure is carried out based on the density functional theory (DFT) implemented in the Vienna ab initio simulation package (VASP) [64], with spin‐orbit coupling included. The projector augmented wave (PAW) method [65] is used for treating the ionic potentials. The generalized gradient approximation (GGA) with the Perdew‐Burke‐Ernzerhof (PBE) [66] realization is adopted for the exchange‐correlation functional. The plane‐wave cutoff energy is set to 400 eV, and the Brillouin zone is sampled by a 12×12×12 mesh. The energy convergence criteria are set as 10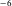 eV for the electronic self‐consistent calculations. Then we use the Wannier90 package [67] to interpolate the DFT Hamiltonian from VASP with the Wannier functions. After transforming the Wannier into the conventional unit cell, we build a slab to simulate the surface spectrum of Bi, as shown in Figure S7d,h, where the surface bands are outlined in red. Then we analyze the irreducible representation (irreps) of the four target surface bands (with spin degree of freedom).

Now we have all the inputs from the first‐principles calculation and we are ready to construct our pristine and moiré surface models. Using those irreps, we first build the symmetry‐inspired minimal tight‐binding model for the Bi surface, from which we calculate the Fermi surface as shown in Figure S8b,c. Then we formulate a moiré band theory to study the moiré Hamiltonian of the Bi interface and use it to compute the trefoil Fermi surface and the Lifshitz transition. It is worth noting that our formulation of moiré Hamiltonian is independent of the commensurability of the lattice, i.e, it can also work in incommensurate structures. See details in the Section S8 (Supporting Information) [46].

### 5.7 Code Availability

The custom code used in this study was developed within the framework of the open‐source MATLAB toolbox TBkit (a computational package for tight‐binding model and DFT post‐processing). The source code is available at https://github.com/LIU‐Binbin/TBkit and has been archived at Zenodo (https://doi.org/10.5281/zenodo.18508250). The specific implementations used for the calculations are provided as part of the package examples: the scripts for constructing the symmetry‐based minimal surface tight‐binding model and the moiré band Hamiltonian can be found in the directory https://github.com/LIU‐Binbin/TBkit/tree/Moire_hamiltonian/example/example10‐Moire_hamiltonian and the scripts for DFT post‐processing from Wannier tight‐binding Hamiltonian are located in the directory https://github.com/LIU‐Binbin/TBkit/tree/Moire_hamiltonian/example/example04‐HR/Wannier_hr_postprocessing.

## Conflicts of Interest

The authors declare no conflict of interest.

## Supporting information


**Supporting File**: adma72540‐sup‐0001‐SuppMat.pdf.

## Data Availability

The data that support the findings of this study are available from the corresponding author upon reasonable request.
