# Supplementary material for: Proximity Induced Magnetic Anisotropy and Trefoil Fermiology in Monolayer FeCl2/Bi(111)
Source: Adv Mater. 2026 Feb 19;38(16):e21534. doi: 10.1002/adma.202521534 (PMC12994352; doi:10.1002/adma.202521534)
Supplement: Supplementary file 1 — Supporting File: adma72540‐sup‐0001‐SuppMat.pdf. [file ADMA-38-e21534-s001.pdf]

Supporting Information for Proximity Induced Magnetic Anisotropy and Trefoil Fermiology at $FeCl_2$/Bi(111) Interface

Shigemi Terakawa Binbin Liu Frank Schindler Titus Neupert Jing-Rong Ji Gabriele Domaine Emily C. McFarlane Daiyu Geng Jiabao Yang Fadi Choueikani Philippe Ohresser Manuel Valvidares Pierluigi Gargiani Craig Polley Gerardina Carbone Mats Leandersson Stuart S. P. Parkin Amilcar Bedoya-Pinto Niels B. M. Schröter**

1 Commensurability of the moiré superstructure

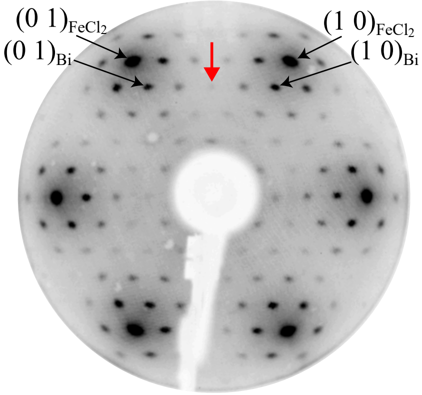

Figure S1: LEED patterns of the 1.0-ML $FeCl_2$ film on Bi(111) ($E_p = 40$ eV). The red arrow indicates one of the diffraction spots corresponding to the moiré superstructure.

Figure S1 shows a high-contrast low-energy electron diffraction (LEED) pattern of the 1.0-ML $FeCl_2$ film on Bi(111). All the LEED spots corresponding to the $(5 \times 5)_{FeCl_2} = (4 \times 4)_{Bi}$ superstructure including higher-order diffraction away from the (1 0) and (0 1) spots are as sharp as those of the Bi(111) surface, indicating that the heterostructure consists of commensurate (5 × 5) $FeCl_2$(0001) and (4 × 4) Bi(111) lattices.

2 Reproducibility of the moiré superstructure

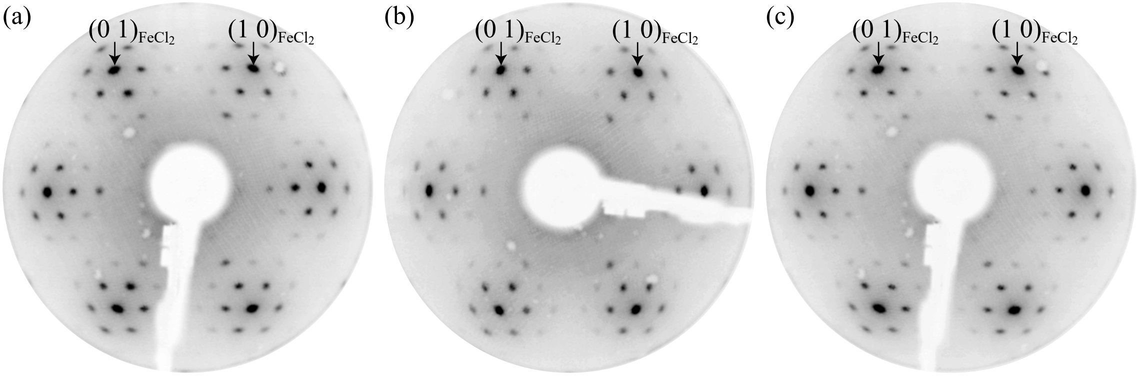

Figure S2: LEED patterns of the three different monolayer $FeCl_2$ films on Bi(111) separately grown for (a) STM, (b) XMCD, and (c) ARPES measurements ($E_p = 46$ eV).

Figure S2 shows LEED patterns of three different monolayer $FeCl_2$/Bi(111) samples separately grown for scanning tunneling microscopy (STM), X-ray magnetic circular dichroism (XMCD), and angle-resolved photoelectron spectroscopy (ARPES) measurements, demonstrating the reproducibility of the moiré superstructure.

3 Magnetic anisotropy energy

We calculate the magnetic anisotropy energy (E_{MAE}), which is defined as $E_{\mathrm{MAE}} = E_{\parallel} - E_{\perp}$. $E_{\parallel}$ and $E_{\perp}$ represent the energy with the magnetization along the in-plane and the out-of-plane directions, respectively. The negative (positive) E_{MAE} value represents the in-plane (out-of-plane) magnetic anisotropy.
In general, there are two main contributions to the E_{MAE}. One is the magnetocrystalline anisotropy energy (E_{MCA}) induced by the spin-orbit coupling and the other is the magnetic shape anisotropy energy (E_{MSA}) originating from the dipole-dipole interaction. The total magnetic anisotropy energy is given by $E_{\mathrm{MAE}} = E_{\mathrm{MCA}} + E_{\mathrm{MSA}}$. The E_{MSA} (= $E_{\mathrm{dipole}\parallel} - E_{\mathrm{dipole}\perp}$) is calculated according to the energy of the magnetic dipole-dipole interaction [1]:

$$E_{\mathrm{dipole}} = \frac{1}{2}\frac{\mu_0}{4\pi}\sum_{i\neq j}^{N}\frac{1}{r_{ij}^3}\left[\boldsymbol{M}_i\cdot\boldsymbol{M}_j - \frac{3}{r_{ij}^2}\left(\boldsymbol{M}_i\cdot\boldsymbol{r}_{ij}\right)\left(\boldsymbol{M}_j\cdot\boldsymbol{r}_{ij}\right)\right]. \tag{1}$$

Here, $\boldsymbol{M}_i$ is the local magnetic moment at an i site, $\boldsymbol{r}_{ij}$ is the vector connecting i and j sites, μ_0 is the vacuum permeability. We set the cutoff value of r_{ij} as 1000 Å, and used the experimentally determined total magnetic moment as the magnitude of $\boldsymbol{M}_i$ and the lattice parameters of the bulk $FeCl_2$.
The E_{MCA} (= $E_{\mathrm{SOC}\parallel} - E_{\mathrm{SOC}\perp}$) is expressed as

$$E_{\mathrm{MCA}} = -\frac{\xi}{4\mu_{\mathrm{B}}}\left(m_{\mathrm{orb}\parallel} - m_{\mathrm{orb}\perp}\right), \tag{2}$$

where $m_{\mathrm{orb}\parallel}$ and $m_{\mathrm{orb}\perp}$ are the orbital magnetic moments along the in-plane and out-of-plane directions, respectively, and ξ is the spin-orbit coupling constant ($\xi = 400$ cm^{-1} for Fe^{2+}) [1, 2, 3, 4].

4 Modified Arrott-plot analysis

Figure S3 shows the modified Arrott plots for eight two-dimensional (2D) and three-dimensional (3D) models including mean-field, Ising, XY and Heisenberg models [5, 6, 7]. M and H correspond to the XMCD intensity and magnetic field, respectively.

5 Polarization-dependence of interface states

Backfolded bands observed by ARPES can be either attributed to initial state effects or umklapp-scattering effects in photoemission final states [8, 9]. In order to distinguish the two effects, we measured the interface states of the monolayer $FeCl_2$/Bi(111) with incident light of $h\nu = 30$ eV having two different polarizations. In Figure S4, we compare the constant energy maps and the band dispersions measured with (a) linear horizontal (LH) and (b) linear vertical (LV) polarizations. It should be noted that the crossing point of the interface bands I in Figure S4 is located at a lower binding energy than that of Figure 5(a) probably due to beam damage after long-term measurement. Figure 5(a) shows a constant energy map measured with the LH polarization at a binding energy of 70 meV. The I_1 band forming the inner corner of the star centered at $\overline{\Gamma}_0$ is strongly observed with the LH polarization. The spectral weight drastically changes with the LV polarization. The intensity of the contours in the first moiré mini surface Brillouin zone (SBZ) and the backfolded I_2 band is stronger than the original star centered at $\overline{\Gamma}_0$ and I_1 band, respectively. This intensity reversal is also seen in the metallic bands at around $k_{\parallel} = 0.2$ Å^{-1} (the bottom panels of Figure S4). Moreover in Figure 5(d), the contours corresponding to the stars centered at

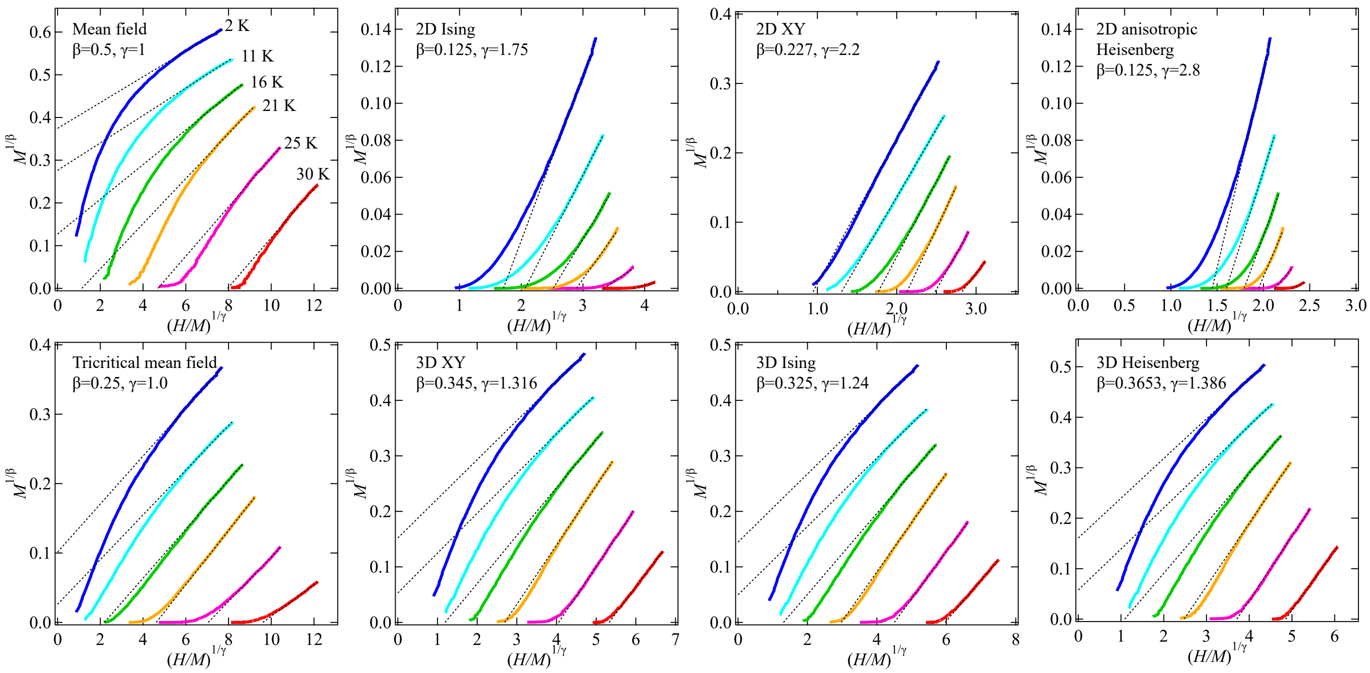

Figure S3: Modified Arrott-plot analysis for the XMCD magnetization curves of the 1.0-ML $FeCl_2$ film on Bi(111) in the GI geometry. M and H correspond to the XMCD intensity and magnetic field, respectively. The black dotted lines represent linear fits to the data in the magnetic field range of 4–6 T.

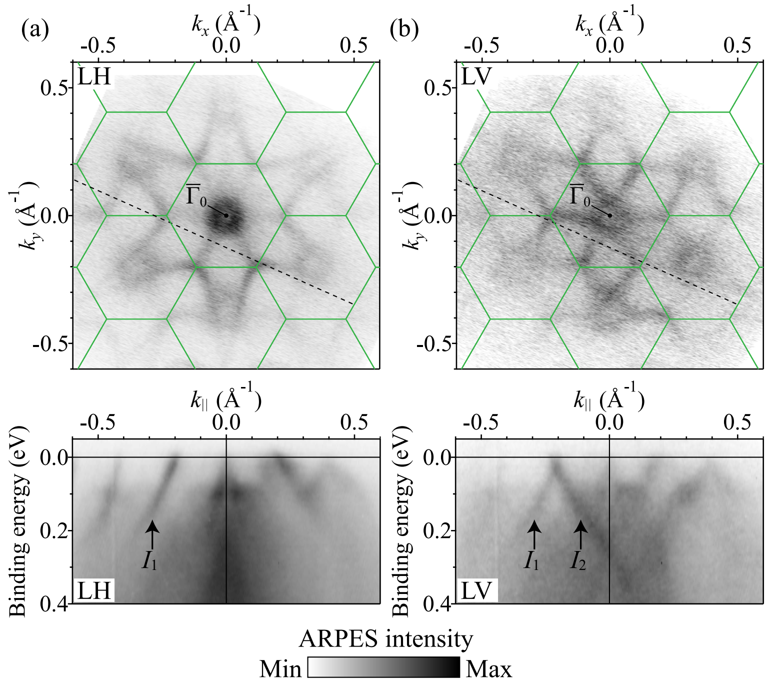

Figure S4: (top) Constant energy maps at a binding energy of 70 meV and (bottom) band dispersions of the 1.0-ML $FeCl_2$ on Bi(111) measured at 77 K and $h\nu = 30$ eV with (a) LH and (b) LV polarizations. The energy window of the constant energy maps was 40 meV. The green solid lines represent the moiré mini-SBZs. The band dispersions in the bottom panels were measured along the dotted lines in the top panels.

the fourth-nearest-neighbor $\overline{\Gamma}_\mathrm{m}$ points from $\overline{\Gamma}_0$ are still strongly observed as denoted by the thick black curves in the schematic. Therefore, we conclude that the initial state effect is the dominant source of the moiré-induced replica bands.

6 Interface states measured at low temperature

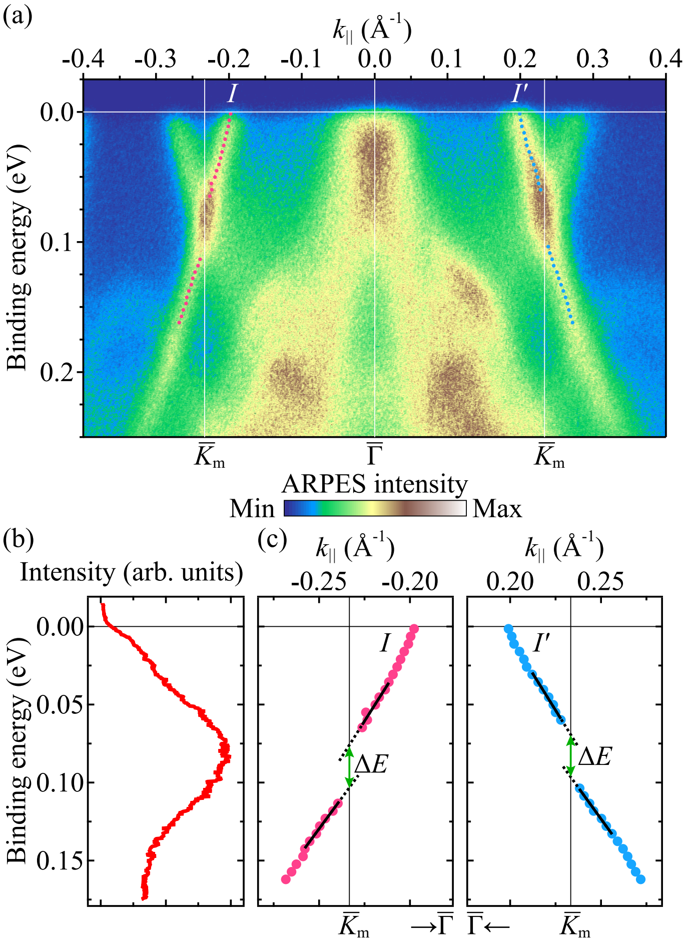

Figure S5: (a) ARPES band dispersion along $\overline{K}_\mathrm{m}$–$\overline{\Gamma}$–$\overline{K}_\mathrm{m}$ of the 1.0-ML $FeCl_2$ film on Bi(111) obtained with He Iα ($h\nu$ = 21.2 eV) at 35 K. The dispersions of the main branches I and I' determined from the peak positions of the momentum distribution curves are plotted using the red and blue circles in (a) and (c). (b) Energy distribution curve at the $\overline{K}_\mathrm{m}$ point ($k_{||}$=−0.233 Å^{-1}). (c) Linear fit to the dispersions of the I and I' bands on the left and right sides of the $\overline{K}_\mathrm{m}$ point. The solid lines indicate the points used for the linear fitting and the dotted lines are the extrapolation toward the $\overline{K}_\mathrm{m}$ point.

Figure S5(a) shows the band dispersion near the Fermi level of the monolayer $FeCl_2$ on Bi(111) measured at 35 K. The features of the interface states observed at room temperature (Figure 5(a)) become clearer. The crossing bands exhibit an anticrossing behavior at the $\overline{K}_\mathrm{m}$ point, although the gap is unclear in the energy distribution curve at the $\overline{K}_\mathrm{m}$ point (Figure S5(b)). In order to evaluate the gap size, we determined the peak positions of the I and I' bands from the momentum distribution curves, which are shown by the red and blue circles in Figure S5(a) and (c). Note that we exclude the points near the $\overline{K}_\mathrm{m}$ points because the peaks corresponding to the I band and the replica bands are not well separated. From the linear fit of the data on the left and right sides of the $\overline{K}_\mathrm{m}$ point, the anticrossing gap ΔE is estimated to be ∼30 meV.

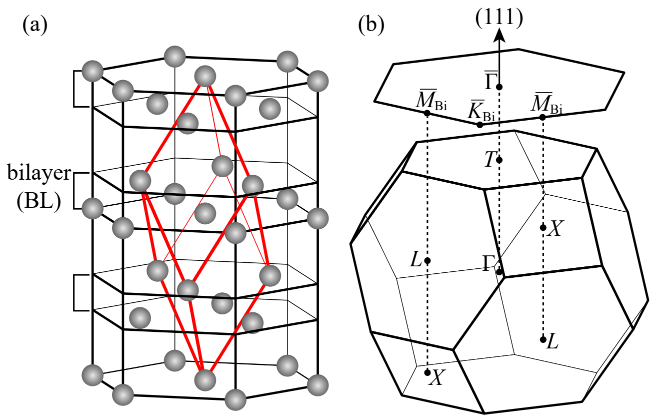

Figure S6: (a) Atomic structure of bulk Bi. The black and red lines indicate the conventional hexagonal unit cell and the primitive rhombohedral unit cell, respectively. (b) Bulk and surface Brillouin zones of Bi(111).

7 Structure of Bi

Figure S6(a) shows the atomic structure of bulk bismuth (Bi). The primitive rhombohedral unit cell indicated by the red lines contains three bilayers (BLs) of Bi. The bulk Brillouin zone (BZ) and (111) surface Brillouin zone (SBZ) are presented in Figure S6(b).

8 Theoretical modeling of the moiré Bi surface

8.1 General Formalism

8.1.1 Lattice geometry conventions

The (111) surface of Bi forms a hexagonal lattice [10]. The lattice vectors are obtained from the in-plane lattice vectors of the conventional hexagonal unit cell of bulk Bi. In our dimensionless conventions, we fix them as

$$\boldsymbol{a}_1 = \begin{pmatrix} 1 \\ 0 \end{pmatrix}, \quad \boldsymbol{a}_2 = \frac{1}{2}\begin{pmatrix} -1 \\ \sqrt{3} \end{pmatrix}. \tag{3}$$

We take the surface normal to be oriented along the z-direction, so that the first vector entry in Equation (3) corresponds to the x-direction and the second entry corresponds to the y-direction. Correspondingly, the reciprocal lattice vectors satisfying $\boldsymbol{b}_i \cdot \boldsymbol{a}_j = 2\pi\delta_{ij}$ read

$$\boldsymbol{b}_1 = 2\pi\begin{pmatrix} 1 \\ \frac{1}{\sqrt{3}} \end{pmatrix}, \quad \boldsymbol{b}_2 = 2\pi\begin{pmatrix} 0 \\ \frac{2}{\sqrt{3}} \end{pmatrix}. \tag{4}$$

Under these conventions, the three inequivalent high-symmetry points $\overline{\Gamma}$, $\overline{M}$, and $\overline{K}$ of the SBZ have coordinate representations

$$\overline{\boldsymbol{\Gamma}} = \boldsymbol{0} = \begin{pmatrix} 0 \\ 0 \end{pmatrix}, \quad \overline{\boldsymbol{M}} = \frac{\boldsymbol{b}_1}{2} = \begin{pmatrix} \pi \\ \frac{\pi}{\sqrt{3}} \end{pmatrix}, \quad \overline{\boldsymbol{K}} = \frac{1}{3}(2\boldsymbol{b}_1 - \boldsymbol{b}_2) = \begin{pmatrix} \frac{4\pi}{3} \\ 0 \end{pmatrix}. \tag{5}$$

The $\overline{K}$ point preserves C_{3z} symmetry. The high-symmetry points $\overline{M}'$ and $\overline{M}''$ are obtained from $\overline{M}$ by applying C_{3z} rotations, and the remaining high-symmetry point $\overline{K}'$ is obtained from $\overline{K}$ by applying time-reversal symmetry.

8.1.2 Formulation of the surface tight-binding model without moiré potential

To model the Bi(111) surface, we work on a spinful electronic tight-binding (TB) model inspired and derived from first-principles density functional theory (DFT) calculations. The surface model extends finitely in z-direction (perpendicular to the surface), and is periodic in x, y-directions (parallel to the surface). The general Hamiltonian reads

$$H_0 = \sum_{\boldsymbol{k}} \sum_{i,j} \sum_{s,s'} [h(\boldsymbol{k})]_{is,js'} \, c^{\dagger}_{\boldsymbol{k},i,s} c_{\boldsymbol{k},j,s'}, \tag{6}$$

where we denote the momenta parallel to the surface as $\boldsymbol{k} = (k_x, k_y)^{\mathrm{T}}$, the orbital degree of freedom as i, j and the spin orientation by $s, s' =\uparrow, \downarrow$. The summation over $\boldsymbol{k}$ is restricted to the first SBZ. The reciprocal lattice forms a hexagonal structure with reciprocal lattice vectors given in Equation (4). The crystal structure of the system is symmetric under C_{3z}-rotation as well as mirror M_x-reflection, therefore, satisfying

$$[H_0, C_{3z}] = [H_0, M_x] = 0. \tag{7}$$

The representation matrices for these symmetries are given by

$$C_{3z} c^{\dagger}_{\boldsymbol{k},i,s} C_{3z}^{-1} = \sum_{j,s'} [D(C_{3z})]_{js',is} \, c^{\dagger}_{C_{3z}\boldsymbol{k},j,s'}, \tag{8}$$

$$M_x c^{\dagger}_{\boldsymbol{k},i,s} M_x^{-1} = \sum_{j,s'} [D(M_x)]_{js',is} \, c^{\dagger}_{M_x\boldsymbol{k},j,s'}. \tag{9}$$

The solutions to the single-particle Hamiltonian H_0 allow us to define the energy band basis

$$c^{\dagger}_{\boldsymbol{k},n} = \sum_{i,s} u_{is;n}(\boldsymbol{k}) c^{\dagger}_{\boldsymbol{k},i,s}, \tag{10}$$

where n is an integer labeling the band index. The Schrödinger equation in the band basis reads

$$\sum_{j,s'} [h(\boldsymbol{k})]_{is,js'} \, u_{js';n}(\boldsymbol{k}) = \epsilon_n(\boldsymbol{k}) u_{is;n}(\boldsymbol{k}). \tag{11}$$

Here, the u_n and ϵ_n are interpreted as the nth eigenfunction and eigenvalue of the Hamiltonian h. This allows us to rewrite the Hamiltonian in the energy band basis as

$$H_0 = \sum_{\boldsymbol{k}} \sum_{n} \epsilon_n(\boldsymbol{k}) c^{\dagger}_{\boldsymbol{k},n} c_{\boldsymbol{k},n}. \tag{12}$$

The representation matrices for C_{3z} as well as M_x can also be expressed in the energy band basis. We derive the case for C_{3z}. Using Equation (10), we can write

$$C_{3z} c^{\dagger}_{\boldsymbol{k},n} C_{3z}^{-1} = \sum_{i,s} u_{is;n}(\boldsymbol{k}) \; C_{3z} c^{\dagger}_{\boldsymbol{k},i,s} C_{3z}^{-1} = \sum_{i,j,s,s'} [D(C_{3z})]_{js',is} \; u_{is;n}(\boldsymbol{k}) \; c^{\dagger}_{C_{3z}\boldsymbol{k},j,s'}. \tag{13}$$

Expressing the fermionic operator again in the band basis, we get

$$c^{\dagger}_{C_{3z}\boldsymbol{k},j,s'} = \sum_{n'} u^{*}_{js';n'}(C_{3z}\boldsymbol{k}) \; c^{\dagger}_{C_{3z}\boldsymbol{k},n'}. \tag{14}$$

Finally we obtain

$$\begin{aligned} C_{3z} c^{\dagger}_{\boldsymbol{k},n} C_{3z}^{-1} &= \sum_{n',i,j,s,s'} u^{*}_{js';n'}(C_{3z}\boldsymbol{k}) [D(C_{3z})]_{js',is} \; u_{is;n}(\boldsymbol{k}) \; c^{\dagger}_{C_{3z}\boldsymbol{k},n'} \\ &= \sum_{n'} [\overline{D}(C_{3z})]_{n',n} \; c^{\dagger}_{C_{3z}\boldsymbol{k},n'}, \end{aligned} \tag{15}$$

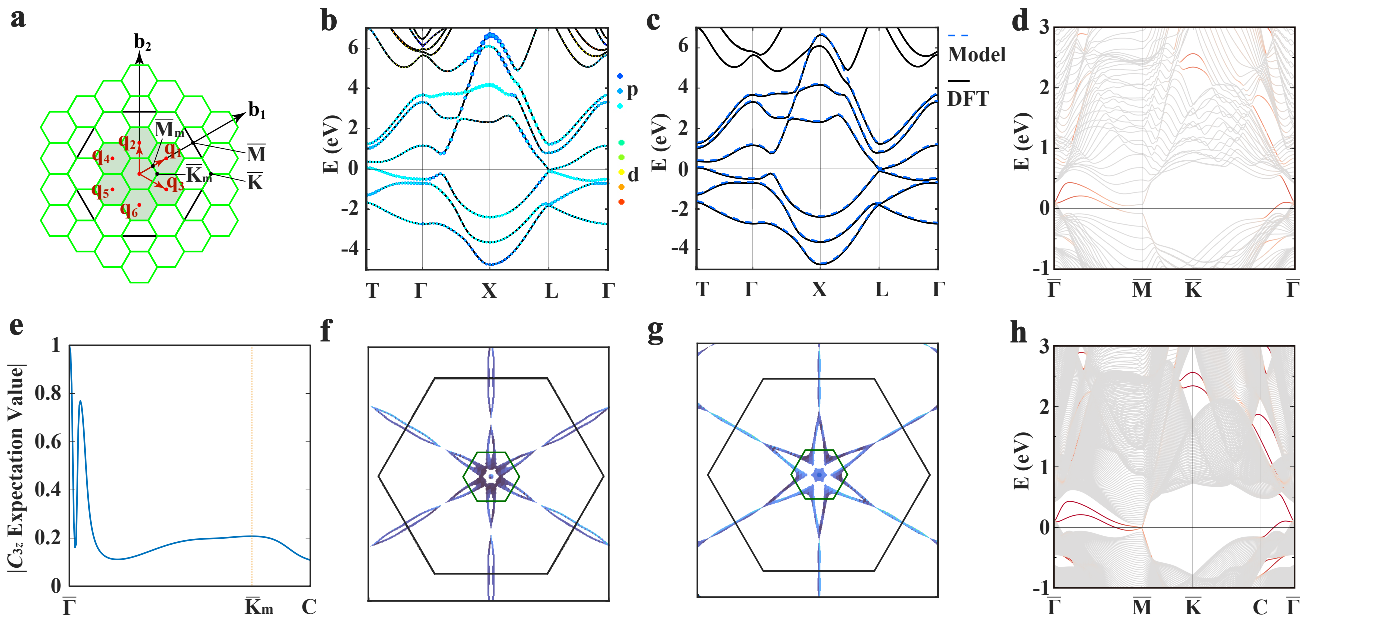

Figure S7: **DFT study of Bi. a** Sketch of SBZ of Bi(111) (black) and moiré mini-SBZ (green). The moiré reciprocal vectors connecting nearest-neighbor mini-SBZs with the first mini-SBZ (shaded in green) are highlighted in red. **b**, **c** The bulk bands of Bi. **b** The bulk bands and orbital character of Bi. The orbital components of the bands are labeled by different colors, with the cold colors corresponding to all three p orbitals ($p_{x,y,z}$) and the warm colors to the five d orbitals. The bands around the Fermi level are primarily p orbitals, which are the basis for our Wannier TB model. **c** The band dispersions from the Wannier TB model and DFT. The Wannier TB model reproduces the DFT bands with high precision. **d**, **h** The band dispersions of the Bi slabs along high symmetry lines. The bulk bands are colored in gray and the surface bands are highlighted in red. **d** corresponds to 18-BL thickness, which approximately agrees with the thickness of the Bi film in our experiment. **h** shows a converged surface band for a slab with 90-BL thickness, where the states at the $\overline{M}$ point become degenerate. **e** The expectation value of the threefold rotation symmetry $|\langle C_{3z}\rangle_{\boldsymbol{k}}|$ along a high symmetry line $\overline{\Gamma}$ to $\overline{K}$. This calculation is done with the lower surface band intersecting the Fermi energy. The surface band is only exposed in the gap from $\overline{\Gamma}$ to the critical point C, and merges into the bulk between C and $\overline{K}$. **f**, **g** Constant energy contours of the Bi slab with the 18-BL thickness from the Wannier TB model. The thin star in **f** is found at a chemical potential of 0.110 eV and the fatter star in **g** at a chemical potential of 0.09 eV. The black and green hexagons refer to the original SBZ and moiré mini-SBZ.

with

$$[\overline{D}(C_{3z})]_{n',n} = \sum_{i,j,s,s'} u^*_{js';n'}(C_{3z}\boldsymbol{k})[D(C_{3z})]_{js',is}u_{is;n}(\boldsymbol{k}). \tag{16}$$

Similarly, the mirror symmetry M_x satisfies the following expression

$$[\overline{D}(M_x)]_{n',n} = \sum_{i,j,s,s'} u^*_{js';n'}(M_x\boldsymbol{k})[D(M_x)]_{js',is}u_{is;n}(\boldsymbol{k}). \tag{17}$$

8.1.3 Formulation of the surface tight-binding model with moiré potential

The presence of a moiré potential can be treated similarly to the Bistritzer-MacDonald model of twisted bilayer graphene [11]. This model effectively realizes hopping on a momentum space lattice formed by moiré reciprocal lattice vectors. From the experimental observations, the moiré mini-SBZ (mBZ) is also found to be oriented along the same axes as the SBZ of the Bi(111) surface given in Equation (4). We denote the moiré reciprocal lattice vectors as $\boldsymbol{b}_{M1} = \gamma\boldsymbol{b}_1$, $\boldsymbol{b}_{M2} = \gamma\boldsymbol{b}_2$, where the γ is the ratio between the moiré BZ and the original BZ. They form the moiré reciprocal lattice $\boldsymbol{Q} = \mathbb{Z}\boldsymbol{b}_{M1} + \mathbb{Z}\boldsymbol{b}_{M2}$ (see Figure S7a). We define the momenta connecting neighboring mBZs as

$$\boldsymbol{q}_1 = \boldsymbol{b}_{M1}, \quad \boldsymbol{q}_2 = \boldsymbol{b}_{M2}, \tag{18}$$

from which we can derive other nearest neighbours (NN) reciprocal moiré lattice vectors such as $\boldsymbol{q}_3 = \boldsymbol{b}_{M1} - \boldsymbol{b}_{M2}$. Neglecting higher-order harmonics, we consider a moiré potential composed of only these NN

non-vanishing moiré lattice vectors,

$$V_{i,j,s,\boldsymbol{r}} = \sum_{l=1}^{6} \sum_{\eta=\pm} e^{i\eta \boldsymbol{q}_l \boldsymbol{r}} V_{i,j,s,\eta \boldsymbol{q}_l}. \tag{19}$$

Our treatments here work not only in a commensurate but also in an incommensurate structural geometry. The moiré potential is non-magnetic, so that it preserves spin s, but it may couple different orbitals i and j. We neglect an overall constant contribution $V^{(0)}_{i,j,s,\boldsymbol{r}} = V^{(0)}_{i,j,s}$, as such a term can be absorbed into the definition of H_0 in Equation (6). We then form the moiré Hamiltonian that acts locally at the position $\boldsymbol{r}$ on internal degrees of freedom i, j and spin s,

$$H_V = \sum_{i,j,s,\boldsymbol{r}} V_{i,j,s,\boldsymbol{r}} c^\dagger_{\boldsymbol{r},i,s} c_{\boldsymbol{r},j,s}. \tag{20}$$

Fourier transforming to momentum space using

$$c^\dagger_{\boldsymbol{r},i,s} = \frac{1}{\sqrt{\mathcal{V}}} \sum_{\boldsymbol{k}} e^{i\boldsymbol{k}\boldsymbol{r}} c^\dagger_{\boldsymbol{k},i,s}, \tag{21}$$

where $\mathcal{V}$ is the total number of original (not moiré) unit cells in the lattice, results in

$$\begin{aligned} H_V &= \sum_{i,j,s,\boldsymbol{k},\boldsymbol{k}'} \left[\frac{1}{\mathcal{V}} \sum_{\boldsymbol{r}} \sum_{l=1}^{6} \sum_{\eta=\pm} e^{i(\boldsymbol{k}-\boldsymbol{k}'+\eta\boldsymbol{q}_l)\boldsymbol{r}} V_{i,j,s,\eta\boldsymbol{q}_l} \right] c^\dagger_{\boldsymbol{k},i,s} c_{\boldsymbol{k}',j,s} \\ &= \sum_{i,j,s,\boldsymbol{k},\boldsymbol{k}'} \sum_{l=1}^{6} \sum_{\eta=\pm} \left[\delta_{\boldsymbol{k},\boldsymbol{k}'-\eta\boldsymbol{q}_l} V_{i,j,s,\eta\boldsymbol{q}_l} \right] c^\dagger_{\boldsymbol{k},i,s} c_{\boldsymbol{k}',j,s}. \end{aligned} \tag{22}$$

Transforming into the band basis using Equation (10) yields

$$H_V = \sum_{\boldsymbol{k}} \sum_{n,n'} \sum_{l=1}^{6} \sum_{\eta=\pm} \underbrace{\left(\sum_{i,j,s} u^*_{is;n}(\boldsymbol{k}) V_{i,j,s,\eta\boldsymbol{q}_l} u_{js;n'}(\boldsymbol{k}+\eta\boldsymbol{q}_l) \right)}_{V(\boldsymbol{k})_{\eta\boldsymbol{q}_l,n,n'}} c^\dagger_{\boldsymbol{k},n} c_{\boldsymbol{k}+\eta\boldsymbol{q}_l,n'}. \tag{23}$$

Finally, we restrict the momentum $\boldsymbol{k}$ to the mBZ and introduce the moiré reciprocal lattice vectors $\boldsymbol{Q}$, $c^\dagger_{\boldsymbol{k}\in\mathrm{BZ},n} \to c^\dagger_{\boldsymbol{k}\in\mathrm{mBZ},\boldsymbol{Q},n}$. Combined with the kinetic part of Equation (12), we can then write the moiré Hamiltonian as

$$H_M = H_0 + H_V = \sum_{\boldsymbol{k}\in\mathrm{mBZ}} \sum_{\boldsymbol{Q},\boldsymbol{Q}'} \sum_{n,n'} h^{(m)}_{\boldsymbol{Q}n,\boldsymbol{Q}'n'}(\boldsymbol{k})\; c^\dagger_{\boldsymbol{k},\boldsymbol{Q},n} c_{\boldsymbol{k},\boldsymbol{Q}',n'}, \tag{24}$$

with

$$h^{(m)}_{\boldsymbol{Q}n,\boldsymbol{Q}'n'}(\boldsymbol{k}) = \epsilon_n(\boldsymbol{k}+\boldsymbol{Q}) \delta_{\boldsymbol{Q},\boldsymbol{Q}'} \delta_{n,n'} + \sum_{l=1}^{6} \sum_{\eta=\pm} \delta_{\boldsymbol{Q},\boldsymbol{Q}'-\eta\boldsymbol{q}_l} V(\boldsymbol{k})_{\eta\boldsymbol{q}_l,n,n'}, \tag{25}$$

where n denotes the bands $\epsilon_n(\boldsymbol{k})$ without moiré potential, the summation over $\boldsymbol{k}$ is restricted to the mBZ and $V_{\eta\boldsymbol{q}_l,n,n'}$ is the moiré potential. In practice, we just consider the $\boldsymbol{Q}$ summing over the mBZs in the origin and its six nearest-neighbours as shown in Figure S7(a), and the moiré coupling in-between them. The action of the C_{3z} and M_x symmetries acting on the creation operator in the moiré band basis according to

$$C_{3z} c^\dagger_{\boldsymbol{k},\boldsymbol{Q},n} C_{3z}^{-1} = \sum_{\boldsymbol{Q}',n'} [\tilde{D}(C_{3z})]_{\boldsymbol{Q}'n',\boldsymbol{Q}n}\; c^\dagger_{C_{3z}\boldsymbol{k},\boldsymbol{Q}',n'}, \tag{26}$$

$$M_x c^\dagger_{\boldsymbol{k},\boldsymbol{Q},n} M_x^{-1} = \sum_{\boldsymbol{Q}',n'} [\tilde{D}(M_x)]_{\boldsymbol{Q}'n',\boldsymbol{Q}n}\; c^\dagger_{M_x\boldsymbol{k},\boldsymbol{Q}',n'}. \tag{27}$$

The representation matrices follow as

$$[\tilde{D}(C_{3z})]_{\boldsymbol{Q}'n',\boldsymbol{Q}n} = \delta_{\boldsymbol{Q}',C_{3z}\boldsymbol{Q}}[\overline{D}(C_{3z})]_{n',n}, \tag{28}$$

$$[\tilde{D}(M_x)]_{\boldsymbol{Q}'n',\boldsymbol{Q}n} = \delta_{\boldsymbol{Q}',M_x\boldsymbol{Q}}[\overline{D}(M_x)]_{n',n}. \tag{29}$$

The overline denotes the representation matrix in the band basis of the Hamiltonian H_0 without moiré potential.

8.2 First-principles and symmetry inspired minimal models for the pristine and moiré Bi

We next elaborate in detail the first-principles calculations we performed for the Bi surface electronic structure, as well as the minimal surface TB model for the pristine Bi and the moiré band model for the $FeCl_2$/Bi heterostructure.

8.2.1 DFT calculation of Bi: bulk and surface

The Bi crystal lies in the space group No. 166 with two Bi atoms per primitive unit cell with a rhombohedral shape (see Figure S6(a)). Our first-principles calculations (Figure S7(b)) suggest that the bands around the Fermi level are primarily composed of $p_{x,y,z}$ orbitals. We therefore build a Wannier TB model including the p orbitals with spin-orbit coupling. This Wannier Hamiltonian model fits the dispersions of the DFT bands from −4 to 6 eV with good precision. It also captures the intrinsic higher-order topology of bulk Bi [12].

We build a slab with 18 BLs of Bi, which approximately corresponds to the thickness of the Bi film in the experiment (6 nm). We construct the (111) surface of Bi with our Wannier Hamiltonian in a conventional unit cell and calculate the surface spectrum of the slab using the Wannier TB model and obtain Figure S7(d). The red bands indicate that their real space projections are on the surface. On the other hand, the gray bands are projected mostly in the bulk. Each visible band is actually two-fold degenerated due to the replica of two surfaces of the slab preserving the presence of time-reversal and inversion symmetries. Correspondingly, there are four bands (including spin) near the Fermi level. The lower two bands form the observed moiré crossing and star-shaped Fermi surface.

To proceed, we analyze the crystalline symmetry irreducible representations (irreps) of the surface bands and then build an effective minimal surface model from the irreps. The irreps of the surface bands at $\overline{\Gamma}$ (in red) are a mixture of one-dimensional (1D) representation $\overline{\mathrm{GM}}_{4-7}$ around the Fermi level and a 2D representation $\overline{\mathrm{GM}}_8$ at higher energies. See Table S1 for a full list of irreps and symmetry characters. The irreps are calculated from the expectation value of the corresponding symmetry operators using Irvsp[13].

8.2.2 Minimum TB model for the Bi surface

Now we construct the Hamiltonian H_0 referred to in Sec. 8.1. We build this model from the DFT-derived symmetry irreps within a Wannier TB basis. Bulk Bi lies in the space group No. 166. However, when considering a freestanding Bi slab exposing the (111) surface, the symmetry group is changed to space group No. 164 with point group symmetry $\mathcal{D}_{3d}$ at the origin of the unit cell. The generators can be chosen as C_{3z}, C_{2x}, and a mirror M_x.

The surface bands have the 1D irreducible representation of $\overline{\mathrm{GM}}_4$ ($\overline{\mathrm{GM}}_5$), $\overline{\mathrm{GM}}_6$ ($\overline{\mathrm{GM}}_7$) at the $\overline{\Gamma}$ point. The parentheses stand for the degenerated time-reversal counterparts. According to the irreps of the $\mathcal{D}_{3d}$ double point group, we choose to represent the symmetries as

$$C_{3z}^I = -\sigma_0 \hat{c}_{3z}, \quad M_x^I = -i\sigma_z \hat{m}_x, \quad T = i\sigma_y \mathcal{K}\hat{I}, \tag{30}$$

[1] The notations of the irreps are according to: (1) C.J. Bradley and A.P. Cracknell, The Mathematical Theory of Symmetry in Solids. Oxford: Clarendon Press (1972), (2) R.S. Mulliken, Phys. Rev. 43, 279-302 (1933). (3) A. P. Cracknell, B. L. Davies, S. C. Miller and W. F. Love, Kronecher Product Tables, 1, General Introduction and Tables of Irreducible Representations of Space Groups. New York: IFI/Plenum (1979), for the GM point.

Table S1: **Irreducible Representation and Character Table of the Double Point Group $\bar{3}$m (No. 20).** The first three columns are different notations of the irreps [1]. The rest of the columns are symmetry operations in the conjugacy classes and their characters. Specifically, they are C_1 : 1, C_2 : $3^+{}_{001}, 3^-001$, C_3 : $2_{1-10}, 2_{120}, 2_{210}$, C_4 : d_1, C_5 : ${}^d3^+{}_{001}, {}^d3^-_{001}$, C_6 : ${}^d2_{1-10}, {}^d2_{120}, {}^d2_{210}$, C_7 : -1, C_8 : $-3^+{}_{001}, -3^-_{001}$, C_9 : $m_{1-10}, m_{120}, m_{210}$, C_{10} : d_{-1}, C_{11} : $d\_3^+{}_{001}, {}^d-3^-001$, C_{12} : ${}^dm_{1-10}, {}^dm_{120}, {}^dm_{210}$.

(1)	(2)	(3)	C_1	C_2	C_3	C_4	C_5	C_6	C_7	C_8	C_9	C_{10}	C_{11}	C_{12}
$GM_1{}^+$	A_{1g}	$GM_1{}^+$	1	1	1	1	1	1	1	1	1	1	1	1
GM_1^-	A_{1u}	GM_1^-	1	1	1	1	1	1	−1	−1	−1	−1	−1	−1
$GM_2{}^+$	A_{2g}	$GM_2{}^+$	1	1	−1	1	1	−1	1	1	−1	1	1	−1
GM_2^-	A_{2u}	GM_2^-	1	1	−1	1	1	−1	−1	−1	1	−1	−1	1
$GM_3{}^+$	E_g	$GM_3{}^+$	2	−1	0	2	−1	0	2	−1	0	2	−1	0
GM_3^-	E_u	GM_3^-	2	−1	0	2	−1	0	−2	1	0	−2	1	0
$GM_4{}^+$	${}^2\overline{E}_g$	$\overline{GM}_4$	1	−1	-i	−1	1	i	1	−1	-i	−1	1	i
$GM_5{}^+$	${}^1\overline{E}_g$	$\overline{GM}_5$	1	−1	i	−1	1	-i	1	−1	i	−1	1	-i
GM_4^-	${}^2\overline{E}_u$	$\overline{GM}_6$	1	−1	-i	−1	1	i	−1	1	i	1	−1	-i
$GM_5{}^-$	${}^1\overline{E}_u$	$\overline{GM}_7$	1	−1	i	−1	1	-i	−1	1	-i	1	−1	i
$GM_6{}^+$	$\overline{E}_{1g}$	$\overline{GM}_8$	2	1	0	−2	−1	0	2	1	0	−2	−1	0
GM_6^-	$\overline{E}_{1u}$	$\overline{GM}_9$	2	1	0	−2	−1	0	−2	−1	0	2	1	0

where operators with hat, $\hat{c}_{3z}$, $\hat{m}_x$ and $\hat{I}$, act on momentum space, and the $\hat{I} = \hat{m}_x\hat{m}_y$. The eigenvalues of mirror symmetry $\pm i$, which follow from $M_x^2 = -1$, imply that the bands are two-fold degenerate along the M_x-preserving lines in the BZ. Note that in the experiment, where Bi is put onto the substrates, C_{2x} symmetry is broken. The point group symmetry is consequently reduced from $\mathcal{D}_{3d}$ to C_{3v}, so that we do not include C_{2x} when constructing the symmetrized TB model. Our DFT results suggest that there are other nearby surface bands which has 2D irreps of $\overline{GM}_8$ ($\overline{GM}_9$) that couple to the surface band near the Fermi level. The 2D symmetry representation reads

$$C_{3z}^{II} = \begin{pmatrix} e^{-i\pi/3} & 0 \\ 0 & e^{i\pi/3} \end{pmatrix} \hat{c}_{3z}, \quad M_x^{II} = \pm \begin{pmatrix} 0 & e^{i\pi/3} \\ e^{i2\pi/3} & 0 \end{pmatrix} \hat{m}_x. \tag{31}$$

The M_x^{II} operator exchanges the chiral and antichiral subspaces of C_{3z} (i.e., eigenstates with C_{3z} eigenvalues $\exp(i\pi/3)$ and $\exp(-i\pi/3)$). The symmetry representations for our minimal four-band model are built the direct sum of the two subspaces and read:

$$C_{3z} = (-\sigma_0) \oplus \begin{pmatrix} e^{-i\pi/3} & 0 \\ 0 & e^{i\pi/3} \end{pmatrix} \hat{c}_{3z}, \quad M_x = (-i\sigma_z) \oplus \begin{pmatrix} 0 & e^{i\pi/3} \\ e^{i2\pi/3} & 0 \end{pmatrix} \hat{m}_x, \quad T = (i\sigma_y) \oplus (i\sigma_y)\mathcal{K}\hat{I}. \tag{32}$$

With these symmetry representations, we can construct a symmetrized four-band TB Hamiltonian H_0 for the surface states, which has the form

$$H_0(\boldsymbol{k}) = \begin{pmatrix} h^I(\boldsymbol{k}) & h_c(\boldsymbol{k}) \\ h_c(\boldsymbol{k})^\dagger & h^{II}(\boldsymbol{k}) \end{pmatrix}, \tag{33}$$

where $h^I(\boldsymbol{k})$ and $h^{II}(\boldsymbol{k})$ stand for the Hamiltonians acting on the bands whose symmetry representations were labeled by I and II, respectively. The details of the surface model are described in 8.2.5. The

matrix $h_c(\boldsymbol{k})$ represents a symmetry-preserving coupling term that mixes the two subspaces. We here only consider the nearest-neighbor hoppings, which can already give a decent fitting to the dispersion of the surface bands and the star-shaped Fermi surface of both the pristine Bi and the moiré $FeCl_2$/Bi interface. The Fermi surfaces calculated from DFT Bi slab corresponding to thinner and relatively fatter hexagonal stars are shown in Figures S7(f) and S7(g), respectively. The star-shaped Fermi surfaces calculated from our minimal surface model are shown in Figures S8(b) and S8(c).

8.2.3 Derivation and estimation of the moiré potential

The moiré potential with first-order harmonic approximation can be written as

$$H_V = \sum_{i,j,s,\boldsymbol{r}} \sum_{l=1}^{6} \sum_{\eta=\pm} e^{i\eta \boldsymbol{q}_l \boldsymbol{r}} V_{i,j,s,\eta\boldsymbol{q}_l} c^{\dagger}_{\boldsymbol{r},i,s} c_{\boldsymbol{r},j,s}, \tag{34}$$

where we have used the Equation (19).
Depending on the stacking configuration of the $FeCl_2$/Bi heterostructure, the moiré potential can have different levels of symmetries. Here, we adopt the highest possible symmetries to continue our analysis, which includes C_{3z}, M_x and T symmetry. The C_{3z} symmetry acts on the H_V as

$$\begin{aligned}
C_{3z} H_V C_{3z}^{-1} &= \sum_{i,j,s,\boldsymbol{r}} \sum_{l=1}^{6} \sum_{\eta=\pm} e^{i\eta C_{3z}\boldsymbol{q}_l \boldsymbol{r}} V_{i,j,s,\eta C_{3z}\boldsymbol{q}_l} \sum_{k,s'} [D(C_{3z})]_{k,i,s'} c^{\dagger}_{\boldsymbol{r},k,s'} \sum_{l,s'} [D(C_{3z})]^{*}_{l,j,s'} c_{\boldsymbol{r},l,s'} \\
&= \sum_{i,j,s,\boldsymbol{r}} \sum_{l=1}^{6} \sum_{\eta=\pm} e^{i\eta \boldsymbol{q}_l \boldsymbol{r}} V_{i,j,s,\eta\boldsymbol{q}_l} \sum_{k,s'} [D(C_{3z})]_{k,i,s'} c^{\dagger}_{\boldsymbol{r},k,s'} \sum_{l,s'} [D(C_{3z})]^{*}_{l,j,s'} c_{\boldsymbol{r},l,s'} \\
&= \sum_{k,l,s',\boldsymbol{r}} \sum_{l=1}^{6} \sum_{\eta=\pm} e^{i\eta \boldsymbol{q}_l \boldsymbol{r}} \sum_{i,j,s} [D(C_{3z})]_{k,i,s'} V_{i,j,s,\eta\boldsymbol{q}_l} [D(C_{3z})]^{\dagger}_{j,l,s'} c^{\dagger}_{\boldsymbol{r},k,s'} c_{\boldsymbol{r},l,s'} \\
&= \sum_{k,l,s',\boldsymbol{r}} \sum_{l=1}^{6} \sum_{\eta=\pm} e^{i\eta \boldsymbol{q}_l \boldsymbol{r}} \{[D(C_{3z})] V_{\eta\boldsymbol{q}_l} [D(C_{3z})]^{-1}\}_{k,l,s'} c^{\dagger}_{\boldsymbol{r},k,s'} c_{\boldsymbol{r},l,s'}.
\end{aligned} \tag{35}$$

The $D(*)$ here is the symmetry representation matrix. Therefore, the constraint that the moiré potential is invariant under C_{3z}, i.e. $C_{3z} H_V C_{3z}^{-1} = H_V$, gives us

$$\{[D(C_{3z})] V_{\eta\boldsymbol{q}_l} [D(C_{3z})]^{-1}\}_{i,j,s} = V_{i,j,s,\eta\boldsymbol{q}_l}. \tag{36}$$

Following the same derivation, we obtain the constraint on the moiré potential from the mirror and time-reversal symmetry as

$$\{[D(M_x)] V_{\eta\boldsymbol{q}_l} [D(M_x)]^{-1}\}_{i,j,s} = V_{i,j,s,\eta\boldsymbol{q}_l}. \tag{37}$$

$$\{[D(T)] V^{*}_{\eta(-\boldsymbol{q}_l)} [D(T)]^{-1}\}_{i,j,s} = V_{i,j,s,\eta\boldsymbol{q}_l}. \tag{38}$$

Together, we find the moiré potential matrix to be

$$V_{\eta\boldsymbol{q}_l} = (v^{I}_{\eta\boldsymbol{q}_l} \oplus v'^{I}_{\eta\boldsymbol{q}_l} \oplus v^{II}_{\eta\boldsymbol{q}_l} \sigma_0) \tag{39}$$

where the $v^{I}_{\eta\boldsymbol{q}_l}$, $v'^{I}_{\eta\boldsymbol{q}_l}$, $v^{II}_{\eta\boldsymbol{q}_l} \in \mathbb{R}$ are the strength the of the moiré potentials which has a form of a diagonal matrix. In the following we assume $v^{I}_{\eta\boldsymbol{q}_l} = v'^{I}_{\eta\boldsymbol{q}_l} = v^{II}_{\eta\boldsymbol{q}_l} = v_0$. Transforming into the band basis following Equation (23), we find that the moiré potential has a neat form

$$V_n(\boldsymbol{k}) = u^{\dagger}_n(\boldsymbol{k}) u_n(\boldsymbol{k} + \eta\boldsymbol{q}_l) v_0, \tag{40}$$

where n is the band index. This means that the elements in the potential matrix are scaled by the overlap between the Bloch states at momenta related by $\eta\boldsymbol{q}_l$. Despite this, we want to emphasize that the I subspace has a much stronger splitting effect compared to the II subspace. This can be understood

as the C_{3z}^I eigenvalue in the I subspace is a constant -1 while the symmetry eigenvalues for the II subspace have destructive interference ($e^{\pm i\pi/3}$) and hence give a small moiré coupling potential. The moiré potential effectively hybridizes and splits different replicas of bands in the mBZ, creating novel topology and connectivity in the Fermi surface. Note that the gap opening physics here is not related to inversion symmetry breaking because there is originally no inversion symmetry in our system.
We examine a Bloch eigenstate $u_n(\boldsymbol{k})$ at $\boldsymbol{K}_m$ (the $\overline{K}$ point in the mBZ) with a band index n. The coupling between the quantum states at the $\boldsymbol{K}_m$ and $\boldsymbol{K}_m + \eta \boldsymbol{q}_l$ can be calculated as

$$u_n^\dagger(\boldsymbol{K}_m) u_n(\boldsymbol{K}_m + \eta \boldsymbol{q}_l) = u_n^\dagger(\boldsymbol{K}_m) C_{3z} u_n(\boldsymbol{K}_m) = \langle C_{3z} \rangle_n(\boldsymbol{K}_m). \tag{41}$$

Note that we enforce a condition that $\eta = -1$ and $l = 2, 3$, so that the states are related by C_{3z} within the mBZ. (Similar for $\boldsymbol{k} = \boldsymbol{K}'_m$.) With Equation 41, one can give a rough estimation of the moiré potential from the C_{3z} eigenvalue for a given band, as we discuss below.
To get some intuition of the moiré potential from the DFT calculations, we examine the moiré potential at $\boldsymbol{K}_m$ ($\boldsymbol{K}'_m$) of the Bi surface band, which can be related to the expectation value of the C_{3z} operator

$$V(\boldsymbol{K}_m)_n = v_0 \langle C_{3z} \rangle_n(\boldsymbol{K}_m). \tag{42}$$

Based on this observation, we calculate the $\langle C_{3z} \rangle_n(\boldsymbol{K}_m)$ along a high-symmetry line from $\overline{\Gamma}$ to $\overline{K}$ in the momentum space for the target surface band. Our slab Wannier TB model has 216 total bands and the 108th band is the surface band near the Fermi level. The norm of the $|\langle C_{3z} \rangle_n(\boldsymbol{K}_m)|$ shown in Figure S7(e) has a value of only one-fifth of the maximum near the $\boldsymbol{K}_m$ point, as opposed to 1 at $\overline{\Gamma}$. In that case, the moiré potential at $\boldsymbol{K}_m$ is small and hence gives rise to the trefoil Fermi surface near $\boldsymbol{K}_m$. We fit our moiré coupling potential to the experiment and get a band splitting of ~27 meV, which corresponds to a parameter of $v_0 = 0.012$ eV.
To understand the essence of our effective model, we need to understand how the coupling of different symmetry subspace gives a dispersed (instead of plain) moiré potential landscape. If we begin with only one C_{3z} subspace with expectation value -1, then we will have a magnified moiré potential as the eigenstates between the $\boldsymbol{K}_m$s always completely overlap with each other, which gives a magnified matrix element. Therefore, a small moiré potential is the result of a mixture of the two C_{3z} subspaces that have different eigenvalues of -1 and $e^{\pm i\pi/3}$. The more the C_{3z}^I subspace gets suppressed, the more the moiré potential gets suppressed. The warping of surface bands creates a strong tweaking in moiré potential, causing an anisotropy in the moiré potential landscape.

8.2.4 Results and Discussion

Figure S8 compares band dispersions and constant-energy contours of a symmetrized minimal surface model and the moiré model with and without a moiré potential. Figures S8(a-c) show the dispersion and the constant energy contours of the pristine Bi surface model. Figures S8(d–f) correspond to the moiré model with a $v_0 = 0.012$ eV. In particular, we plot two distinct phases of the Lifshitz transition that occur in the Fermi surface. Figure S8(e) shows the moiré-folded trefoil Fermi surface arising from the three replicas of the warped Bi surface states. In Figure S8(f), we plot another set of contours at an appropriate hole doping where the three replicas are disentangled and form a Fermi-surface topology distinct from the previous one. A Lifshitz transition relates the two topologically distinct Fermi surfaces. Due to small hybridization, the center of the trefoil is detached from the outliers (the "leaves"), creating a small Fermi pocket, in contrast to the case with no gap due to the absence of a moiré potential (Figure S8(h)). A critical Lifshitz point occurs when the three surfaces touch near the $\overline{K}_m$ point (Figure S8(i)). Note that the $E = 0$ eV in Figure S8 corresponds to the Fermi level determined by the qualitative agreement in the band features and the shape of the Fermi surface between the experiment and calculation.
The small band and the associated momentum splitting due to the moiré potential can fall below the ARPES energy/momentum resolution. However, magnetic-field probes can detect the small central pocket and inter-pocket tunneling via the magnetic breakdown effect and moiré Fermi-surface reconstruction. Under a strong magnetic field, the central small pocket can be connected to the outliers, forming a multiply connected trajectory that features a field-dependent quantum-oscillation frequency. This enables

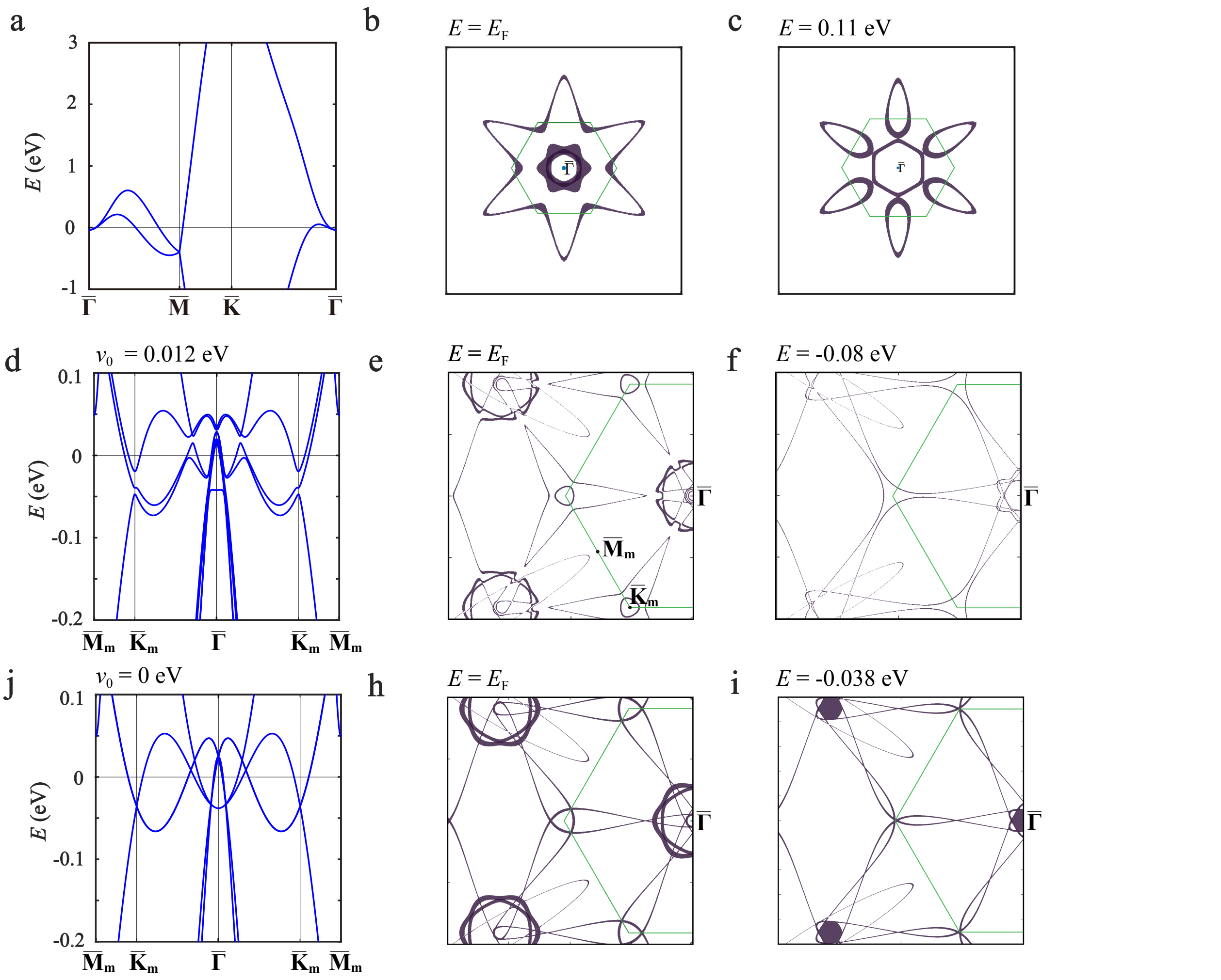

Figure S8: **Symmetrized minimal surface model and moiré calculation. a** The band dispersion of the Bi surface model along high symmetry lines. The constant energy contours at **b** the Fermi level and **c** $E = 0.11$ eV. **d** The electronic structure of the moiré superlattice with $v_0 = 0.012$ eV along a high symmetry line $\overline{M}_m$–$\overline{K}_m$–$\overline{\Gamma}$–$\overline{K}_m$–$\overline{M}_m$ in the mBZ. The gap at $\overline{K}_m$ is around 27 meV. The constant energy contours of the moiré band model at **e** the Fermi level and **f** $E = -0.08$ eV, indicating a Lifshitz transition in the Fermi surface. **j** The electronic structure of the moiré superlattice with $v_0 = 0$ eV. The constant energy contours of the moiré band model at **h** the Fermi level and **i** $E = -0.038$ eV without moiré potential. Figure **i** shows a critical point where the three Fermi surfaces touch at the $\overline{K}_m$ point. The green hexagons indicate the first mBZ.

us a quantitative estimate of the gap. Undergoing Lifshitz transition changes the number and connectivity of pockets near $\overline{K}_m$, alters the quantum-oscillation spectra, and can sharply modulate magnetoresistance.
The trefoil Fermi surface features an exotic moiré fermiology. Unlike graphene on Ir(111) [14], the cone-like band near $\overline{K}_m$ in our system has a different origin; it does not exist in the pristine system but is formed by the band folding. The exotic Lifshitz transition results from the warped (star-shaped) Fermi surface in the original Bi and its interplay with the moiré potential. With our model, we show the Lifshitz transition from the Fermi surfaces, indicating new possibilities via tunable Fermi-surface topology through Lifshitz control. Our theoretical scheme, introduced in the Methods section and the supporting information, can be further applied to study other moiré heterostructure materials.

8.2.5 Details of the Bi surface tight-binding Hamiltonian

Our Hamiltonian is built using the hopping list in Table S2. In three dimensions, the lattice vectors are

$$\boldsymbol{R} = n_1\boldsymbol{a}_1 + n_2\boldsymbol{a}_2 + n_3\boldsymbol{a}_3, \tag{43}$$

where the $\boldsymbol{a}_{1,2,3}$ are lattice vectors. In our case, the $\boldsymbol{a}_3 = \boldsymbol{0}$, since the system is quasi-2D. The TB orbital of site i with spin s in the unit cell labeled by $\boldsymbol{R}$ is denoted as $|\phi_{\boldsymbol{R}i,s}\rangle$, which after transforming into the Bloch-like basis, becomes

$$|\widetilde{\chi}_j^{\boldsymbol{k}}\rangle = \frac{1}{\sqrt{\mathcal{V}}} \sum_{\boldsymbol{R}} e^{i\boldsymbol{k}\cdot\boldsymbol{R}} |\phi_{\boldsymbol{R}i,s}\rangle \,. \tag{44}$$

The matrix element of the Hamiltonian $H_0(\boldsymbol{k})$ is then

$$h(\boldsymbol{k})_{is,js'} = \langle \widetilde{\chi}_{i,s}^{\boldsymbol{k}} | h(\boldsymbol{R}) | \widetilde{\chi}_{j',s'}^{\boldsymbol{k}} \rangle = \sum_{\boldsymbol{R}} e^{i\boldsymbol{k}\cdot\boldsymbol{R}} h_{i,j,s,s'}(\boldsymbol{R}), \tag{45}$$

where the $h_{i,j,s,s'}(\boldsymbol{R})$ is the matrix elements for the real space Hamiltonian, which is listed in the last two columns of Table S2. With the above preparation, we obtain our Hamiltonian H_0 appearing in Equation (6). The parameters used in this paper are $\gamma_1 = -0.50, \gamma_2 = 2.96, \gamma_4 = -0.32, \gamma_5 = 0.66, \gamma_{12} = -1.20$, in unit of eV. γ_2 is the Fermi energy, γ_4 and γ_5 are couplings between two different symmetry subspaces, which play a key role in determining the moiré coupling in the mBZ. The γ_1 affects the first subspace, and the γ_{12} controls only the second subspace. We note that the model parameters are not unique; we here adopt a set that is qualitatively enough to explain the experiments. With these five parameters, we are able to build a minimum TB model describing the Bi surface band structure.

Table S2: The hopping list of the TB Hamiltonian H_0. The first three columns are the lattice vector integers defined in Equation (43). The last two columns are the real and imaginary parts of the real-space Hamiltonian $h_{i,j,s,s'}(\mathbf{R})$ that enter into the Hamiltonian matrix labeled by the orbitals and spins in the fourth and fifth columns.

n_1	n_2	n_3	i, s	j, s'	$\mathrm{Re}[h_{i,j,s,s'}(\mathbf{R})]$	$\mathrm{Im}[h_{i,j,s,s'}(\mathbf{R})]$
-1	-1	0	1	1	γ_1	0
-1	0	0	1	1	γ_1	0
0	-1	0	1	1	γ_1	0
0	0	0	1	1	γ_2	0
0	1	0	1	1	γ_1	0
1	0	0	1	1	γ_1	0
1	1	0	1	1	γ_1	0
-1	-1	0	3	1	γ_4	$-\sqrt{3}\,\gamma_4$
-1	0	0	3	1	γ_5	$-\frac{\sqrt{3}}{3}\gamma_5$
0	-1	0	3	1	$-\gamma_5$	$-\frac{\sqrt{3}}{3}\gamma_5$
0	1	0	3	1	γ_4	$\sqrt{3}\,\gamma_4$
1	0	0	3	1	$-2\,\gamma_4$	0
1	1	0	3	1	0	$\frac{2\sqrt{3}}{3}\gamma_5$
-1	-1	0	4	1	$\frac{\sqrt{3}}{3}\gamma_5$	γ_5
-1	0	0	4	1	$\sqrt{3}\,\gamma_4$	γ_4
0	-1	0	4	1	$-\sqrt{3}\,\gamma_4$	γ_4
0	1	0	4	1	$\frac{\sqrt{3}}{3}\gamma_5$	$-\gamma_5$
1	0	0	4	1	$-\frac{2\sqrt{3}}{3}\gamma_5$	0

1	1	0	4	1	0	-2 γ_4
-1	-1	0	2	2	γ_1	0
-1	0	0	2	2	γ_1	0
0	-1	0	2	2	γ_1	0
0	0	0	2	2	γ_2	0
0	1	0	2	2	γ_1	0
1	0	0	2	2	γ_1	0
1	1	0	2	2	γ_1	0
-1	-1	0	3	2	$-\frac{\sqrt{3}}{3}\gamma_5$	γ_5
-1	0	0	3	2	$-\sqrt{3}\gamma_4$	γ_4
0	-1	0	3	2	$\sqrt{3}\gamma_4$	γ_4
0	1	0	3	2	$-\frac{\sqrt{3}}{3}\gamma_5$	$-\gamma_5$
1	0	0	3	2	$\frac{2\sqrt{3}}{3}\gamma_5$	0
1	1	0	3	2	0	-2 γ_4
-1	-1	0	4	2	γ_4	$\sqrt{3}\gamma_4$
-1	0	0	4	2	γ_5	$\frac{\sqrt{3}}{3}\gamma_5$
0	-1	0	4	2	$-\gamma_5$	$\frac{\sqrt{3}}{3}\gamma_5$
0	1	0	4	2	γ_4	$-\sqrt{3}\gamma_4$
1	0	0	4	2	-2 γ_4	0
1	1	0	4	2	0	$-\frac{2\sqrt{3}}{3}\gamma_5$
-1	-1	0	1	3	0	$-\frac{2\sqrt{3}}{3}\gamma_5$
-1	0	0	1	3	-2 γ_4	0
0	-1	0	1	3	γ_4	$-\sqrt{3}\gamma_4$
0	1	0	1	3	$-\gamma_5$	$\frac{\sqrt{3}}{3}\gamma_5$
1	0	0	1	3	γ_5	$\frac{\sqrt{3}}{3}\gamma_5$
1	1	0	1	3	γ_4	$\sqrt{3}\gamma_4$
-1	-1	0	2	3	0	2 γ_4
-1	0	0	2	3	$\frac{2\sqrt{3}}{3}\gamma_5$	0
0	-1	0	2	3	$-\frac{\sqrt{3}}{3}\gamma_5$	γ_5
0	1	0	2	3	$\sqrt{3}\gamma_4$	$-\gamma_4$
1	0	0	2	3	$-\sqrt{3}\gamma_4$	$-\gamma_4$
1	1	0	2	3	$-\frac{\sqrt{3}}{3}\gamma_5$	$-\gamma_5$
-1	-1	0	3	3	0	γ_{12}
-1	0	0	3	3	0	$-\gamma_{12}$

0	-1	0	3	3	0	-γ_{12}
0	0	0	3	3	γ_2	0
0	1	0	3	3	0	γ_{12}
1	0	0	3	3	0	γ_{12}
1	1	0	3	3	0	-γ_{12}
-1	-1	0	1	4	0	$2\,\gamma_4$
-1	0	0	1	4	-$\frac{2\sqrt{3}}{3}\,\gamma_5$	0
0	-1	0	1	4	$\frac{\sqrt{3}}{3}\,\gamma_5$	γ_5
0	1	0	1	4	-$\sqrt{3}\,\gamma_4$	-γ_4
1	0	0	1	4	$\sqrt{3}\,\gamma_4$	-γ_4
1	1	0	1	4	$\frac{\sqrt{3}}{3}\,\gamma_5$	-γ_5
-1	-1	0	2	4	0	$\frac{2\sqrt{3}}{3}\,\gamma_5$
-1	0	0	2	4	-2 γ_4	0
0	-1	0	2	4	γ_4	$\sqrt{3}\,\gamma_4$
0	1	0	2	4	-γ_5	-$\frac{\sqrt{3}}{3}\,\gamma_5$
1	0	0	2	4	γ_5	-$\frac{\sqrt{3}}{3}\,\gamma_5$
1	1	0	2	4	γ_4	-$\sqrt{3}\,\gamma_4$
-1	-1	0	4	4	0	-γ_{12}
-1	0	0	4	4	0	γ_{12}
0	-1	0	4	4	0	γ_{12}
0	0	0	4	4	γ_2	0
0	1	0	4	4	0	-γ_{12}
1	0	0	4	4	0	-γ_{12}
1	1	0	4	4	0	γ_{12}

9 Computational Methods

Direct computation on the large-scale supercell of moiré Bi surface bands from DFT calculations is difficult, as one has to compute a supercell of $N_{slab} \times N_m \times N_m$ times the original Bi. In our experiment, $N_{slab} = 6$ standing for 18 BLs of Bi, and $N_m = 4$ is induced by the moiré supercell. Note that these numbers can easily grow with increased Bi thickness and additional twisting that enlarges the moiré structure. To mitigate the computational burden, we apply a combined DFT, Wannier function, symmetry and TB method to reduce the dimensions of the Bi surface Hamiltonian, which mitigates the cost of the moire Hamiltonian construction. Our strategy is to construct a minimal symmetry-inspired TB model fitted from our first-principles calculation of the Bi surface, instead of using a naive continuum model around a high-symmetry point. Then we construct the continuous moiré Hamiltonian based on the minimal lattice model. In this way, we achieved a model closer to an *ab initio* calculation of the moiré heterostructure system. It is worth noting that our construction of moiré Hamiltonian is agnostic of the commensurability of the lattice, i.e, it can also work in incommensurate structures. Besides, we are also

able to simulate the global Fermi surfaces in the entire BZ, in addition to the local mBZ behavior. We show the detailed workflows of our moiré calculations in the Methods section in the main manuscript. The symmetry-inspired TB method extends the conventional TB approach by systematically incorporating the crystal symmetries of a material into the construction of the Hamiltonian. Rather than relying solely on fitting parameters or localized orbital intuition, this method uses group theory and symmetry constraints to determine the allowed forms of the hopping terms and onsite energies. This ensures that the resulting model respects the fundamental symmetry operations of the underlying lattice, such as rotations, reflections, and time-reversal symmetry. As a result, the symmetry-inspired TB method provides a more rigorous and physically grounded framework, enabling accurate modeling of complex electronic structures—including topological phases, degeneracies protected by symmetry, and can be used for moiré Hamiltonian construction while retaining computational efficiency.

References

[1] J. Stöhr. Exploring the microscopic origin of magnetic anisotropies with X-ray magnetic circular dichroism (XMCD) spectroscopy, *Journal of Magnetism and Magnetic Materials* **1999**, *200* 470.

[2] G. M. Cole, B. B. Garrett. Atomic and Molecular Spin-Orbit Coupling Constants for 3d Transition Metal Ions, *Inorganic Chemistry* **1970**, *9* 1898.

[3] P. Bruno. Tight-binding approach to the orbital magnetic moment and magnetocrystalline anisotropy of transition-metal monolayers, *Physical Review B* **1989**, *39* 865.

[4] A. S. Dale, S. Yazdani, T. K. Ekanayaka, E. Mishra, Y. Hu, P. A. Dowben, J. W. Freeland, J. Zhang, R. Cheng. Direct observation of the magnetic anisotropy of an Fe(II) spin crossover molecular thin film, *Journal of Physics: Materials* **2023**, *6* 035010.

[5] B. Liu, Y. Zou, L. Zhang, S. Zhou, Z. Wang, W. Wang, Z. Qu, Y. Zhang. Critical Behavior of the Quasi-Two-Dimensional Semiconducting Ferromagnet $CrSiTe_3$, *Scientific Reports* **2016**, *6* 33873.

[6] A. Bedoya-Pinto, J. R. Ji, A. K. Pandeya, P. Gargiani, M. Valvidares, P. Sessi, J. M. Taylor, F. Radu, K. Chang, S. S. Parkin. Intrinsic 2D-XY Ferromagnetism in a van der Waals Monolayer, *Science* **2021**, *374* 616.

[7] Q. H. Wang, A. Bedoya-Pinto, M. Blei, A. H. Dismukes, A. Hamo, S. Jenkins, M. Koperski, Y. Liu, Q. C. Sun, E. J. Telford, H. H. Kim, M. Augustin, U. Vool, J. X. Yin, L. H. Li, A. Falin, C. R. Dean, F. Casanova, R. F. Evans, M. Chshiev, A. Mishchenko, C. Petrovic, R. He, L. Zhao, A. W. Tsen, B. D. Gerardot, M. Brotons-Gisbert, Z. Guguchia, X. Roy, S. Tongay, Z. Wang, M. Z. Hasan, J. Wrachtrup, A. Yacoby, A. Fert, S. Parkin, K. S. Novoselov, P. Dai, L. Balicas, E. J. Santos. The magnetic genome of two-dimensional van der waals materials, *ACS Nano* **2022**, *16* 6960.

[8] C. M. Polley, L. I. Johansson, H. Fedderwitz, T. Balasubramanian, M. Leandersson, J. Adell, R. Yakimova, C. Jacobi. Origin of the π-Band Replicas in the Electronic Structure of Graphene Grown on 4H-SiC(0001), *Physical Review B* **2019**, *99* 115404.

[9] A. J. Graham, H. Park, P. V. Nguyen, J. Nunn, V. Kandyba, M. Cattelan, A. Giampietri, A. Barinov, K. Watanabe, T. Taniguchi, A. Andreev, M. Rudner, X. Xu, N. R. Wilson, D. H. Cobden. Conduction Band Replicas in a 2D Moiré Semiconductor Heterobilayer, *Nano Letters* **2024**, *24* 5117.

[10] H. Mönig, J. Sun, Y. M. Koroteev, G. Bihlmayer, J. Wells, E. V. Chulkov, K. Pohl, P. Hofmann. Structure of the (111) surface of bismuth: Leed analysis and first-principles calculations, *Physical Review B* **2005**, *72* 085410.

[11] R. Bistritzer, A. H. MacDonald. Moiré bands in twisted double-layer graphene, *Proceedings of the National Academy of Sciences* **2011**, *108* 12233.

[12] F. Schindler, Z. Wang, M. G. Vergniory, A. M. Cook, A. Murani, S. Sengupta, A. Y. Kasumov, R. Deblock, S. Jeon, I. Drozdov, H. Bouchiat, S. Guéron, A. Yazdani, B. A. Bernevig, T. Neupert. Higher-order Topology in Bismuth, *Nat. Phys.* **2018**, *14* 918.

[13] J. Gao, Q. Wu, C. Persson, Z. Wang. Irvsp: To obtain irreducible representations of electronic states in the VASP, *Computer Physics Communications* **2021**, *261* 107760.

[14] I. Pletikosić, M. Kralj, P. Pervan, R. Brako, J. Coraux, A. T. N'Diaye, C. Busse, T. Michely. Dirac cones and minigaps for graphene on Ir(111), *Physical Review Letters* **2009**, *102* 056808.
